# Supplementary material for: In-tube micro-pyramidal silicon nanopore for inertial-kinetic sensing of single molecules
Source: Nat Commun. 2024 Jun 15;15:5132. doi: 10.1038/s41467-024-48630-5 (PMC11180207; doi:10.1038/s41467-024-48630-5)
Supplement: Supplementary file 1 — Supplementary Information [file 41467_2024_48630_MOESM1_ESM.pdf]

Supplementary Materials for

In-tube micro-pyramidal silicon nanopore for inertial-kinetic sensing of single molecules

Jianxin Yang[1], Tianle Pan[1], Zhenming Xie[1], Wu Yuan[1]*, Ho-Pui Ho[1]*

[1]Department of Biomedical Engineering, The Chinese University of Hong Kong, Hong Kong SAR, China.

*Corresponding authors. Email: wyuan@cuhk.edu.hk (W.Y.); aaron.ho@cuhk.edu.hk (H.-P.H.).

Main Contents

Supplementary Note 1. Comparison of conventional nanopore fabrication methods with photovoltaic electrochemical etch-stop technique (our method). 4

Supplementary Note 2. Calculations of the mechanical strength of MPSN and a typical silicon nitride pore. 5

Supplementary Note 3. The feedback blockade signals generated by electrokinetic translocation events ($\xi target \neq \xi nanopore$) of BSA and EpCAM in the in-tube device. ..6

Supplementary Note 4. The preparation process in MPSN fabrication. 7

Supplementary Note 5. Etching systems and calibration of silicon sample position during etching. 8

Supplementary Note 6. Measurement of remaining thickness H. 10

Supplementary Note 7. Study the light power dependence of photoinhibition-assisted KOH etching on silicon. 11

Supplementary Note 8. Simulations of the transmitted spectra of MPSN during the etching process. 12

Supplementary Note 9. TEM images and I-V curves of nanopore samples corresponding to the datapoints shown in Figure 2e and f. 14

Supplementary Note 10. MPSN pore size measurement through grayscale analysis. 15

Supplementary Note 11. The repeatability study of the photovoltaic electrochemical etch-stop technique. 17

Supplementary Note 12. Setting of the in-tube nanopore sensing device. 18

Supplementary Note 13. Molecular translocation under different pH values. 19

Supplementary Note 14. Test the stability of MPSN. 20

Supplementary Note 15. Sensing area of MPSN. 21

Supplementary Note 16. Current traces of Au@PEG NPs under different pH values, rotation speeds, and applied voltages, and the dependence of capture rates on molecular concentration and rotation speed. 23

Supplementary Note 17. Compare the capture radius of the inertial-kinetic translocation with electrokinetic translocation in MPSN. 26

Supplementary Note 18. The centrifugal force experienced by molecules at different rotational speeds in MPSN. 28

Supplementary Note 19. Mechanism of centrifugal-based molecular motions in MPSN (a) Diffusion Coefficient $D\beta$. 29

(b) Motion of molecules in capture process (ii) 29

(c) Motion of molecules in translocation-through-nanopore process (iii) 30

(d) Dependence of dwell time on molecular configuration 30

Supplementary Note 20. Current traces for sensing the dissociation antibody-antigen complex and the aggregation of Au@PEG nanoparticles in 15-nm MPSN. 31

Supplementary Note 21. Study the signal characteristics of EpCAM IgG and Au@PEG nanoparticles in trimolecular aggregate. *33*

Supplementary Note 22. Current traces for sensing the dissociation of antibody-antigen complex in 20- and 23-nm MPSN. *34*

Supplementary Note 23. Current traces of Au@PEGs aggregations purified using centrifugation-based separation protocol. *36*

Supplementary References *38*

Supplementary Note 1. Comparison of conventional nanopore fabrication methods with photovoltaic electrochemical etch-stop technique (our method).

Table S1. Conventional nanopore fabrication techniques versus PALE.

Fabrication Methods	Material	Thickness	Pore size	Pore shape	Scalability/repeatability
FIB	SiN[1-5]	< 30 nm	[0.3 nm, 280 nm][1,2,6-8]	Cylindrical	Yes
	SiO_2[8,9]	[0.5 nm, 60 nm]		Cylindrical	
	SiC[6,10]	< 20 nm[6]		Cylindrical	
	2D materials[7,11,12]	around 0.3nm[13]		Cylindrical	
TEM drilling	SiN	< 40 nm[14]	> 0.28 nm [14-19]	Cylindrical	Yes
	MoS_2	around 0.7 nm[17,18]		Cylindrical	
	SiO_2	< 40 nm[14]		Cylindrical	
E-beam lithography	Si	50-100 nm[20,21]	5 -113 nm	Funnel-shaped	Yes
Controlled dielectric breakdown	SiN	>10nm[22]	> 1.1 nm[22-26]	Cylindrical	Yes
	SiO_2	>30nm[23]		Cylindrical	
	HfO_2	>10nm[24]		Cylindrical	
Glass pulling	glass[27-31]	nanopore with over tens micrometers[30]	>11 nm[31]	Funnel-shaped	Yes
Chemical etching	Si[32-34]	nanopore with micro thick membrane[33]	> 8 nm[34]	Funnel-shaped	Yes
Photovoltaic electrochemical etch-stop technique (our method)[35,36]	Si	over tens micrometers	> 4.5 nm	Funnel-shaped	Yes

Supplementary Note 2. Calculations of the mechanical strength of MPSN and a typical silicon nitride pore.

When the nanopore structure is subjected to the centrifugal force f_c exceeding the breaking load f_{load} ($f_c > f_{load}$), the nanopore will be broken[37,38]. The relationship between the breaking load f_{load} and the ultimate compressive strength δ can be described as[37,39]:

$$f_{load} = \frac{2w\delta T^2}{3L} \qquad \text{(S1)}$$

where the ultimate compressive strength δ is decided by the material's property, i.e., 6 GPa for single crystal silicon[37] and 3.4 GPa for Si_3N_4 deposited with PECVD[39]; L is the structural length, such as 30 μm for the MPSN and 70 μm for a typical Si_3N_4 nanopore[40]; w is the structural width, for example, 30 μm for MPSN and 70 μm for a typical Si_3N_4 nanopore[40]; T is the structural thickness, which is 21.25 μm for MPSN and 30 nm for the Si_3N_4 nanopore[40]).

According to Equation S1, the breaking load f_{load} of a typical Si_3N_4 nanopore (w = 70 μm; L = 70 μm; T = 30 nm) and MPSN (w = 30 μm; L = 30 μm; T = 21.25 μm) can be calculated as 2.1 μN and 1.8 N, respectively.

Moreover, the centrifugal force f_c exerted on the nanopore samples can be described as:

$$f_c = m\rho\omega^2 \qquad \text{(S2)}$$

where m is the molecular weight (4.7×10^{-13} kg for the Si_3N_4 nanopore; 3.0×10^{-11} kg for the MPSN); ρ is the nominal rotation radius of the nanopore sample and can be assumed as the distance between the nanopore to the spinning centre of centrifuge, i.e., about 25.6 cm in the experiments; ω is the rotational speed.

According to Equation S1 and S2, the Si_3N_4 nanopore and MPSN are able to maintain the mechanical stability, i.e., $f_c < f_{load}$, at rotation speeds ω below 3.6×10^4 and 3.8×10^6 rpm, respectively.

Supplementary Note 3. The feedback blockade signals generated by electrokinetic translocation events ($\xi_{target} \neq \xi_{nanopore}$) of BSA and EpCAM in the in-tube device.

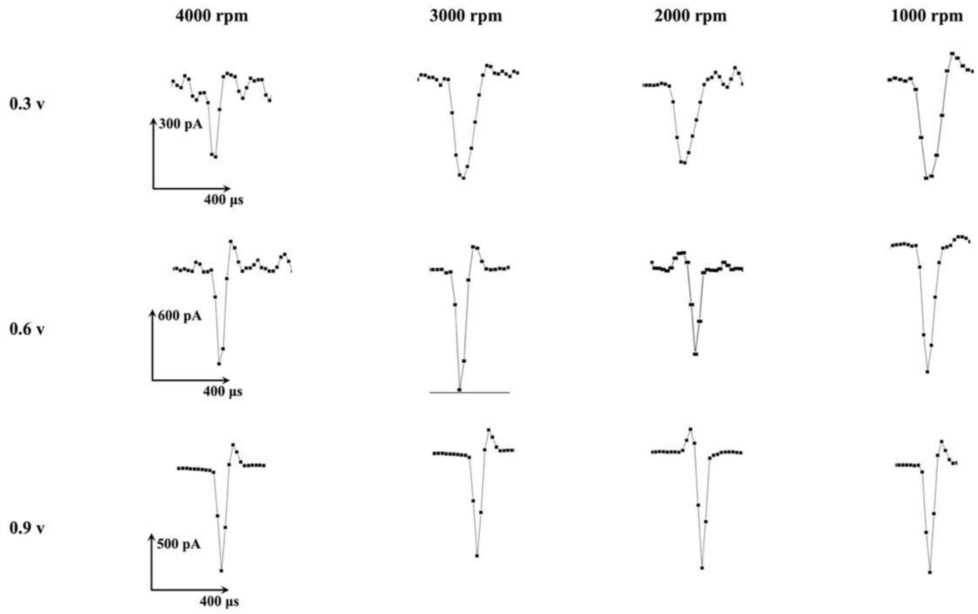

Figure S1. The electrokinetic blockade signals of BSA measured at different rotation speeds (1000 rpm, 2000 rpm, 3000 rpm, and 4000 rpm) and voltages bias (0.3 V, 0.6 V, and 0.9 V) when the pH value of the analyte (i.e., 4.0) wasn't set to induce the balanced state of electrophoretic and electroosmosis effects in MPSN. All signals were acquired at a sampling rate of 50 kHz.

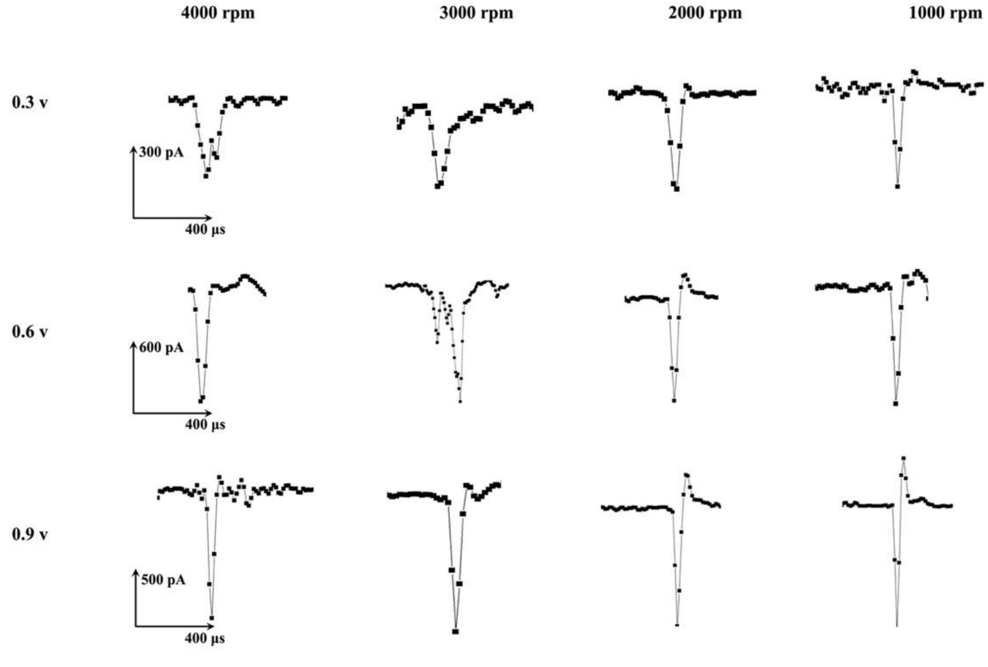

Figure S2. The electrokinetic blockade signals of EpCAM measured at different rotation speeds (1000 rpm, 2000 rpm, 3000 rpm, and 4000 rpm) and voltages bias (0.3 V, 0.6 V, and 0.9 V) when the pH value of the analyte (i.e., 3.4) wasn't set to induce the balanced state of electrophoretic and electroosmosis effects in MPSN. All signals were acquired at a sampling rate of 50 kHz.

Supplementary Note 4. The preparation process in MPSN fabrication.

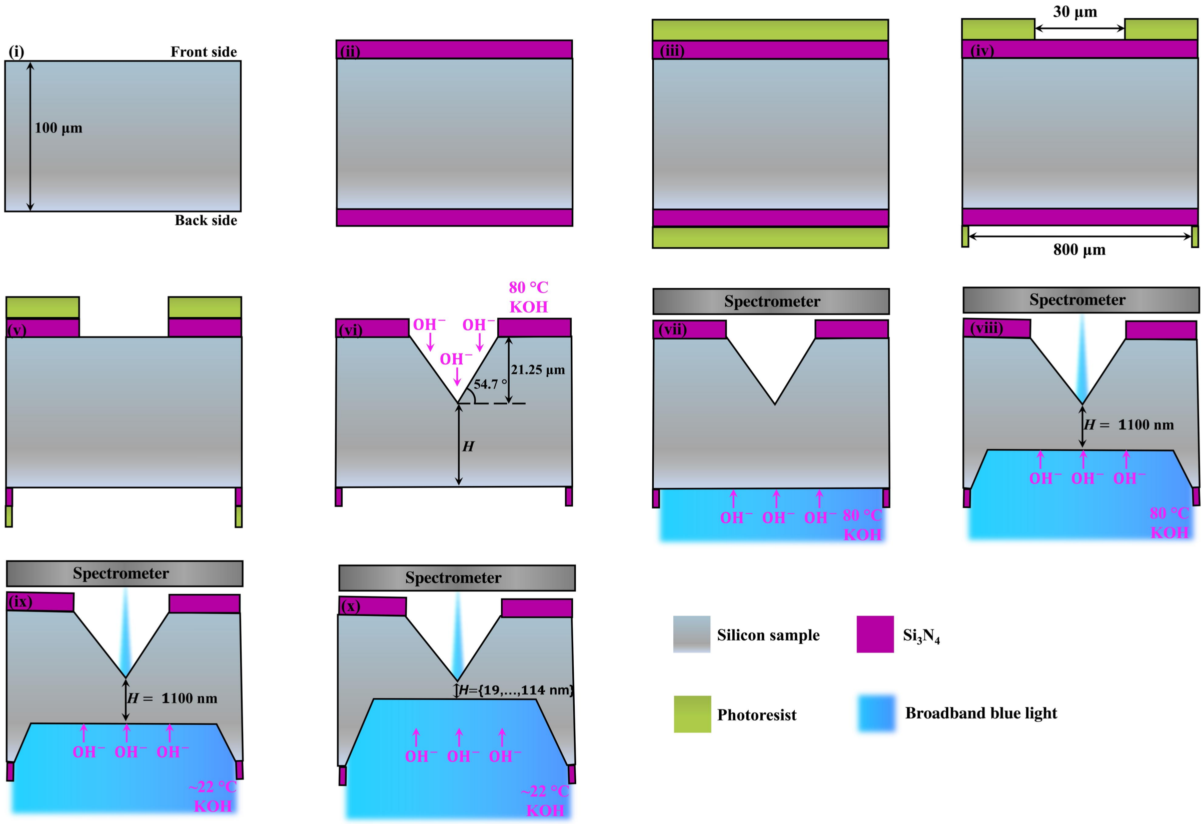

Figure S3. Schematic of the preparation process in MPSN fabrication. (i) 100-μm-thick (1-0-0) silicon wafer for nanopore fabrication; (ii) Deposition of 200-nm Si_3N_4 on two sides of silicon sample; (iii) spin coating of 5-μm positive photoresist (AZ5214E, MicroChemicals) on two sides of silicon sample; (iv) photolithography to transfer two square patterns to photoresist; (v) plasma etching to transfer the two square patterns to Si_3N_4; (vi) KOH etching to fabricate an inverted micro-pyramid structure of about 21.25 μm in depth on the front side; (vii-x) detailed steps of pre-etching process to achieve remaining thickness *H* of 114 to 19 nm. In this process, 80.0 °C KOH is first used to etch the back side of silicon sample at etching rate of about 1.5 μm/min until the remaining thickness reaches about 1100 nm. This procedure is closely monitored using a spectral detection system and the transmission spectral peak is blue shifted to 520 nm when the remaining thickness is 1100 nm. After that, the room-temperature KOH etching process is subsequently performed to achieve a remaining thickness of 114 to 19 nm by closely tracking the transmission spectral peak.

Supplementary Note 5. Etching systems and calibration of silicon sample position during etching.

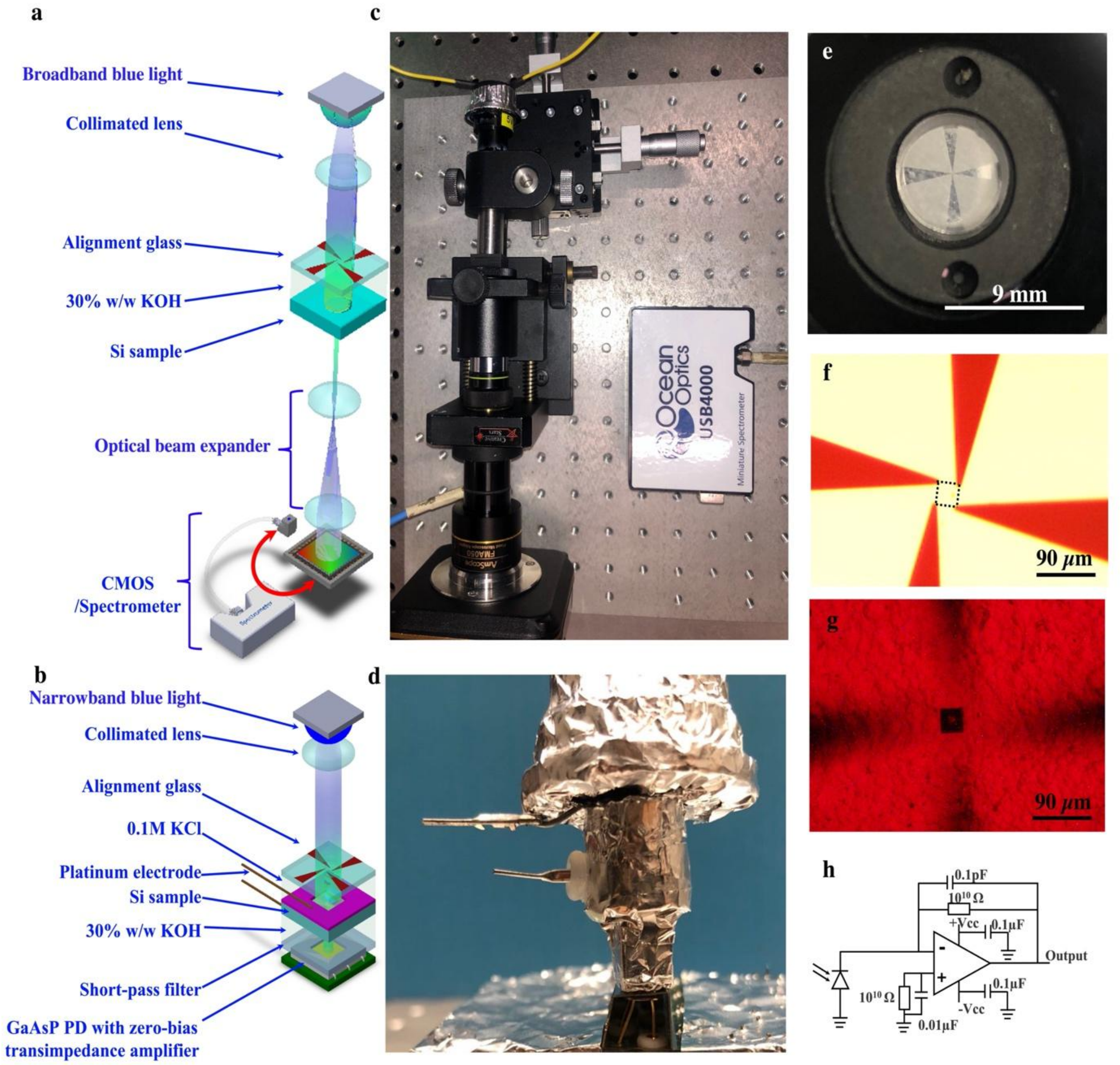

Figure S4. The schematics (a) and images (c) of spectral detection system for monitoring pre-etching process. The schematics of spectral detection system mainly consisting of broadband blue light source, optical alignment system, silicon sample, etchant, spectrometer, and CMOS. The schematic (b) and the image (d) of electro-optical system for performing near-field photoinhibition-assisted electrochemical etching process. The schematics of electro-optical system mainly consisting of narrowband blue light source, photodiode, operational amplifier, and electrical circuit. (e) The image of the quartz glass with alignment pattern. (f) The CMOS image observed by the system without sample, in which the pattern can be observed to be four chrome arrows with their tips spaced 30 μm apart. Four black dot lines connecting the four tips form a square pattern. (g) The CMOS image observed by the system with an etched silicon sample. The sample position is adjusted to align four corners of the front-side pyramid with the tip of the four arrows. (h) The circuit diagram of the photodiode amplifier circuit in the electro-optical system.

In the spectral detection system, 460 nm light (output power: 0.77 W) with a full-width-at-half-maximum (FWHM) bandwidth of 60 nm is incident from back side and collimated with a convex lens (collimated beam size: 8.1 mm). A spectrometer (USB4000, Ocean Optics) with 0.1 nm spectral resolution was located on the front side to monitor the peak position change of transmission spectrum. The wafer was etched from back side by 30% (w/w) KOH at room temperature (~ 22.0 °C), which can real-time measure the etching process by monitoring blue

shift of spectral peak λ_p to long-term monitor the etching process and precisely control the remaining thickness H. Here, the dependence of spectral peak λ_p position on remaining thickness H was aligned by a contact steeper (see Supplementary Note 6).

In the electro-optical system, 460 nm narrowband light (output power: 0.77 W) is incident from front side and collimated with a convex lens (collimated beam size: 8.1 mm), and near-field transmission fringe is distributed at the remaining silicon layer. Reverse photovoltage V_O was generated in the spot because of the directed motion of photogenerated holes and electrons by applying 800 mV forward bias V_p, enabling to regulate the etching rate and area and eventually the pore size of MPSN.

The quartz glass with alignment pattern were fixed in the spectral detection system and electro-optical system as a reference coordinate system for aligning the position of etched sample, in which the pattern is four chrome arrows with their tips spaced 30 μm apart, enabling align the top corners of the bottom of the pyramid structure etched in samples (Fig. S4e-g). The alignment accuracy of the methods was measured as 2 μm.

To further improve the alignment accuracy between position of transmission fringe and of the sample in the electro-optical system, a photodiode circuit (photoelectric sensitivity: 10^9 V/W) was set up on the back side to finely align the sample position by detecting the transmitted intensity. As shown in Fig. S4h, the GaAsP photodiode, whose response is 0.1 A/W at 475 nm, was connected to the inversing input of the transimpedance operational amplifier. The feedback resistance of the amplifier was 10^{10} Ω. The photoelectric sensitivity of the whole platform was expressed as follows:

$$V_{out} = R_\lambda . R_1 . P = 10^9 \text{ V/W} . P \qquad (S3)$$

Output voltage of platform V_{out}, photodiode response R_λ, feedback resistance R_1, and tested optical power P. The sample position is finely tuned to get the V_{out}-H regression curve, enabling to repeat the near-field etching process without bias of the diffraction pattern in the remaining layer.

Supplementary Note 6. Measurement of remaining thickness *H*.

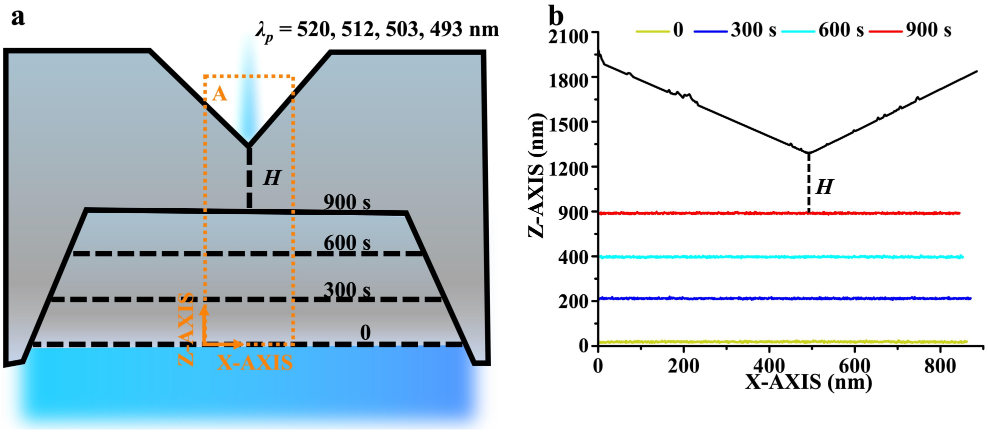

Figure S5. (a) Experimental setup schematic for studying the change in remaining thickness (*H*) during the pre-etching process. The transmission spectral peak (λ_P) blue-shifted to 520 nm, and nanopore's back-side structure profiles were measured every 300 seconds using a profilometer (Tencor Alpha-Step 500 profilometer, KLA-Tencor). The corresponding λ_P values (520, 512, 503, and 493 nm for 0, 300, 600, and 900 seconds) were recorded. (b) Profiles of the front side (black) and back side of MPSN at λ_P = 520 (gold), 512 (blue), 503 (cyan), and 493 (red) nm in the area A in (a), as measured by the profilometer. The origin is set on the backside surface of MPSN at λ_P = 520 nm, and Z-axis represents the height along a cross section of the X-axis.

The tested data can align the relationship between remaining thickness and peak position, which can be fitted as a negative exponential equation, which can be described as: $\lambda_p = -54.0\mathrm{e}^{\frac{1270-H}{2090.67}} + 574.0$.

Supplementary Note 7. Study the light power dependence of photoinhibition-assisted KOH etching on silicon.

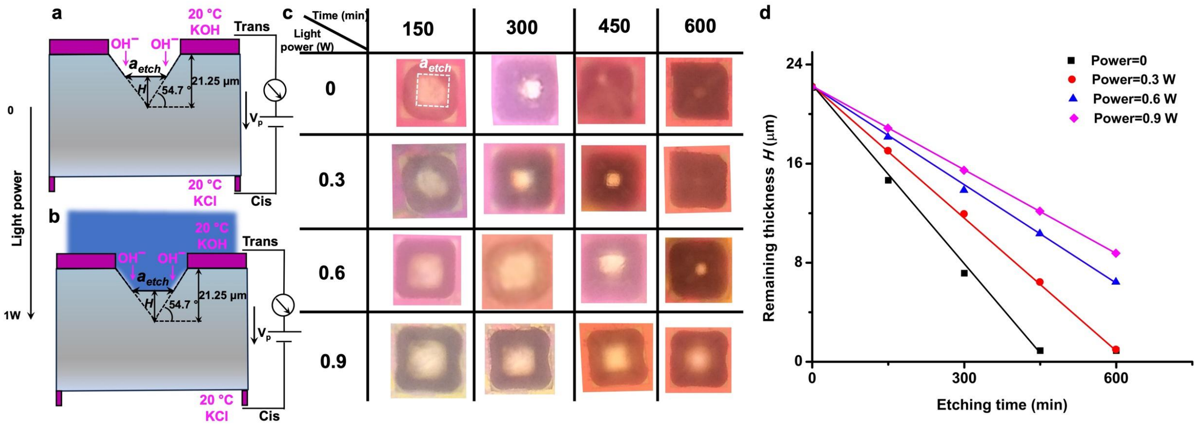

Figure S6. (a and b) Schematics of experimental setting to study the light power dependence of photoinhibition-assisted KOH etching on silicon at room temperature. The remaining thickness H has known linear dependence on square side length a_{etch} measured in the silicon pyramid, i.e., $\frac{2H}{a_{etch}} = tan54.7^{\circ}$. (c) The microscope photographs of etched silicon samples using different etching time and light power. The square sides are labelled with dashed white lines. (d) The dependence of silicon thickness H on the etching time and light power. The fitted lines indicate that the etching rate decreases with the increase of light power. Light power of 0 W, 0.3 W, 0.6 W, and 0.9 W led to etching rates of 54 nm/min (black), 37 nm/min (red), 28 nm/min (blue), and 23 nm/min (purple), respectively.

The photoinhibition-assisted KOH etching technique is also known as photovoltaic electrochemical etch-stop technique and used for micro and nano manufacturing before[35,36]. The mechanism of this technique is that the photovoltage induced by the separation of the photogenerated hole-electron pairs partially offsets the applied potential, resulting in a slower etching rate on light illuminated areas (bright areas) than these on dark areas at the silicon-KOH interface (see Figure 1a).

To illustrate the dependence of light power in this technique, a control experiment has been performed to study the silicon etching rate of KOH at room temperature using 460-nm narrowband light of different light power ranging from 0 to 0.9 W. The light is collimated using a convex lens to achieve a collimated beam size of 8.1 mm on the silicon samples (Figures S6a and b). Figure S6c shows the microscope photographs of etched silicon samples using different etching time and light power. From these photographs, we can further calculate the remaining silicon thickness H by measuring the square side length a_{etch} in the silicon pyramid. The dependence of light power in the photoinhibition-assisted KOH etching technique is shown in Figure. S6d, demonstrating that the etch rate at room temperature decreases with the increase of light power.

Supplementary Note 8. Simulations of the transmitted spectra of MPSN during the etching process.

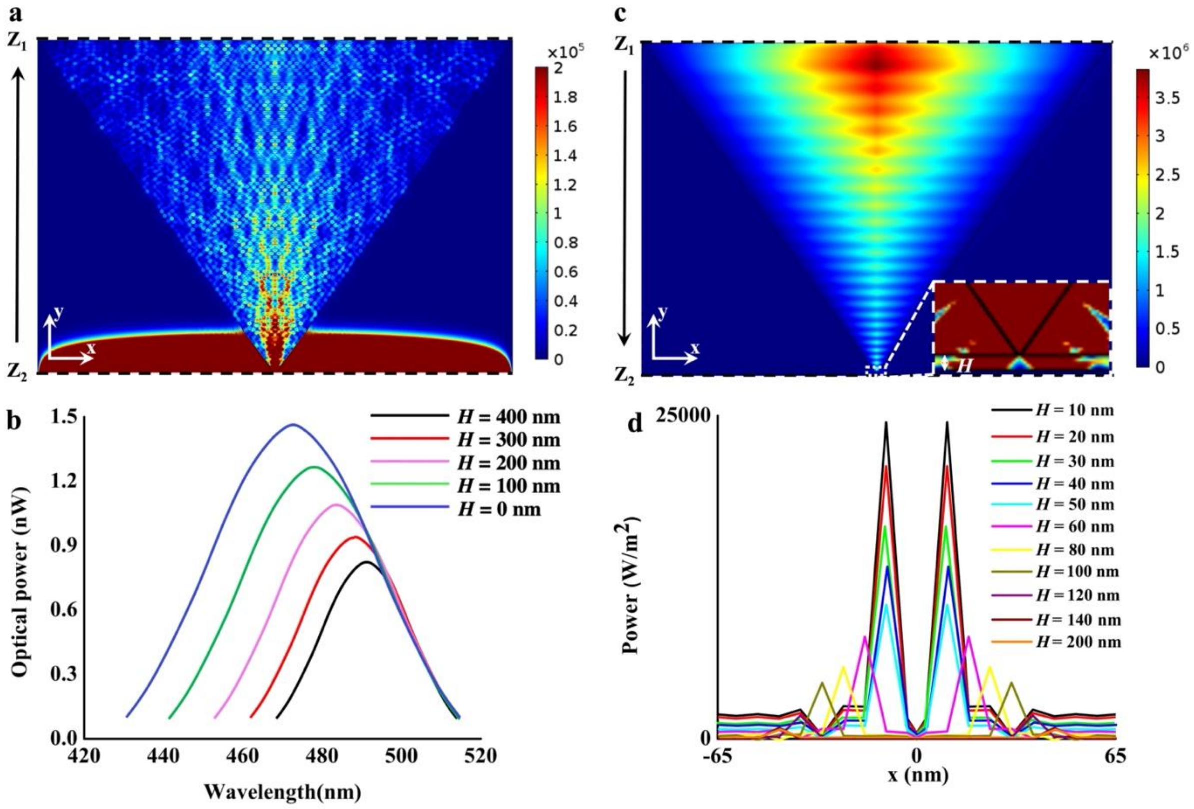

Figure S7. (a) Time averaged Poynting vector <***S***> distribution when light with wavelength λ of 475 nm is incident from the back side boundary Z_2 to the front side boundary Z_1, thickness H of remaining silicon layer is 0. (b) Transmission spectrum in Z_1 for H = 0, 100, 200, 300, and 400 nm when a broadband incident light with peak wavelength = 460 nm and FWHM bandwidth = 60 nm is incident from Z_2. (c) <***S***> distribution when λ_P = 475 nm light is incident from Z_1 at H = 10 nm. Inset shows a ring-shape near-field diffraction pattern in the remaining layer at H = 10 nm. (d) The enlarged distribution curve of <***S***> along x from -65 nm to 65 nm in Z_2 of (c). In the model, x = 0 is set on the central axis.

The micro-pyramid structure was simulated by COMSOL Multiphysics. For the pre-etching process, the simulation results were displayed in the form of optical distribution on a two-dimensional cross section through the pyramid tip, as shown in Fig. S7a. The simulation reflects that peak position of transmitted light blue shifts with the reduce of the thickness H of the remaining layer while light enters the model from the boundary Z_2 (Fig. S7a and b). The distribution of <***S***> in the structure was calculated while monochromatic light incident (Fig. S7a), and then incident wavelength was parametric scanned from 430 nm to 515 nm for simulating the wideband incident light with 120 nm bandwidth, enabling plot corresponding wideband transmission spectrum by space integrating <***S***> of different wavelength along Z_1. The transmission spectrum as function of thickness H was expressed as Fig. S3b. Every point in these curves is representative of integral value of <***S***> collected by a plot as shown in Fig. S7a.

For the near-field etching process, the simulation reveals that the near-field ($T < \lambda/2$) transmission pattern changes with the further decrease of the thickness H from 200 nm to 10 nm while light with λ_P = 475 nm enters the model from the boundary Z_1 (Fig. S7c and d). The spatial distribution shown in Fig. S7c was obtained, whose near-field transmission pattern on the boundary Z_2 shows characteristic features of focusing on around 130 nm central area and increasing with reduce of H (Fig. S7d). A bimodal diffraction distribution was displayed in the

centre of dozens of nanometers, and spatial distance between the two focusing areas trends to be decreased with the decrease of H. It is noted that the two-peaks distance is around 45 nm while H is around 100 nm, which has been confirmed by the diameter of ring-shaped unetched area in Fig. 2.

Supplementary Note 9. TEM images and *I-V* curves of nanopore samples corresponding to the datapoints shown in Figure 2e and f.

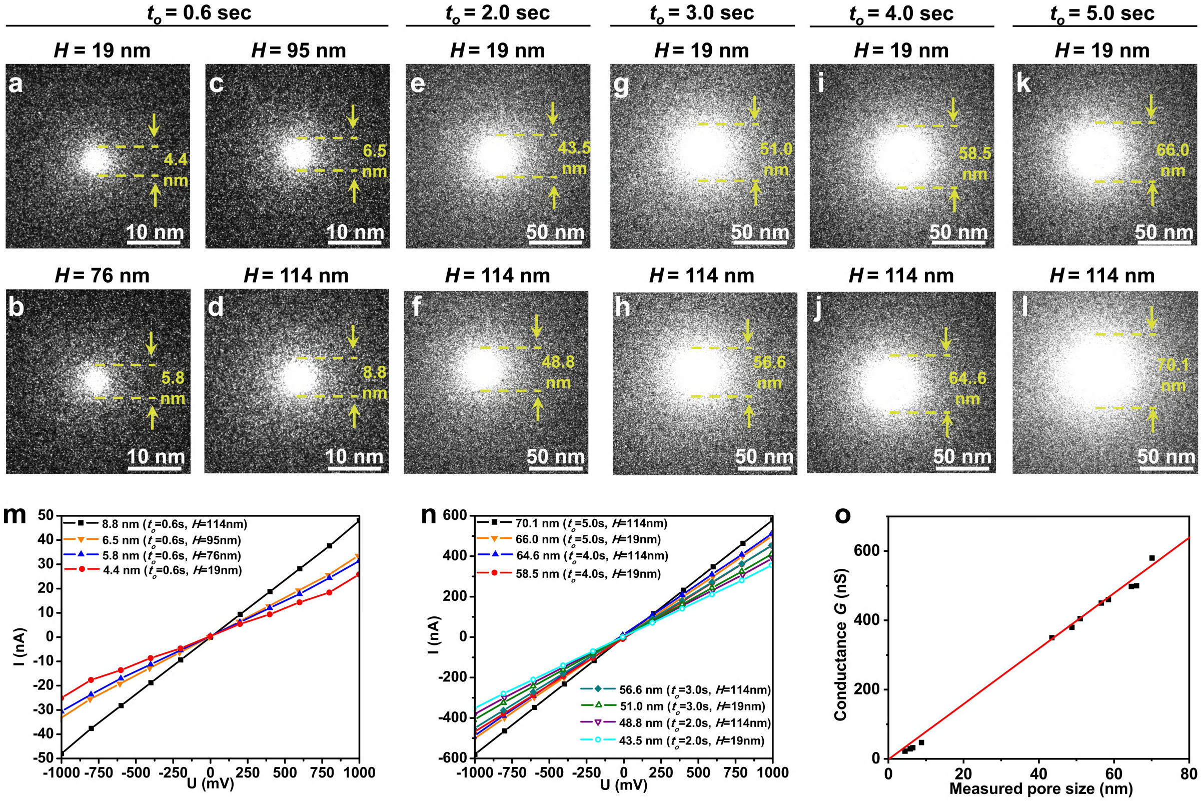

Figure S8. (a-l) Back-view TEM images of MPSNs fabricated using different remaining thickness *H* (or irradiation time t_i) and over-etching time t_o. (m and n) *I-V* curves of each MPSN shown in (a-l), measured in 1M KCl solution using Ag/AgCl electrodes. (o) Measured conductance G (black dots, extracted from the *I-V* curves in m and n) plotted against the pore sizes measured from TEM images. The fitted line (in red) follows the proposed conductance-pore size equation i.e., $G = \frac{\pi d \sigma}{3.68}\sqrt{\frac{90-\theta}{90}}$[41,42], where d, σ, and θ are the pore size of nanopore, the solution conductivity, and the slanted sidewall angel of 54.7°, respectively.

Supplementary Note 10. MPSN pore size measurement through grayscale analysis.

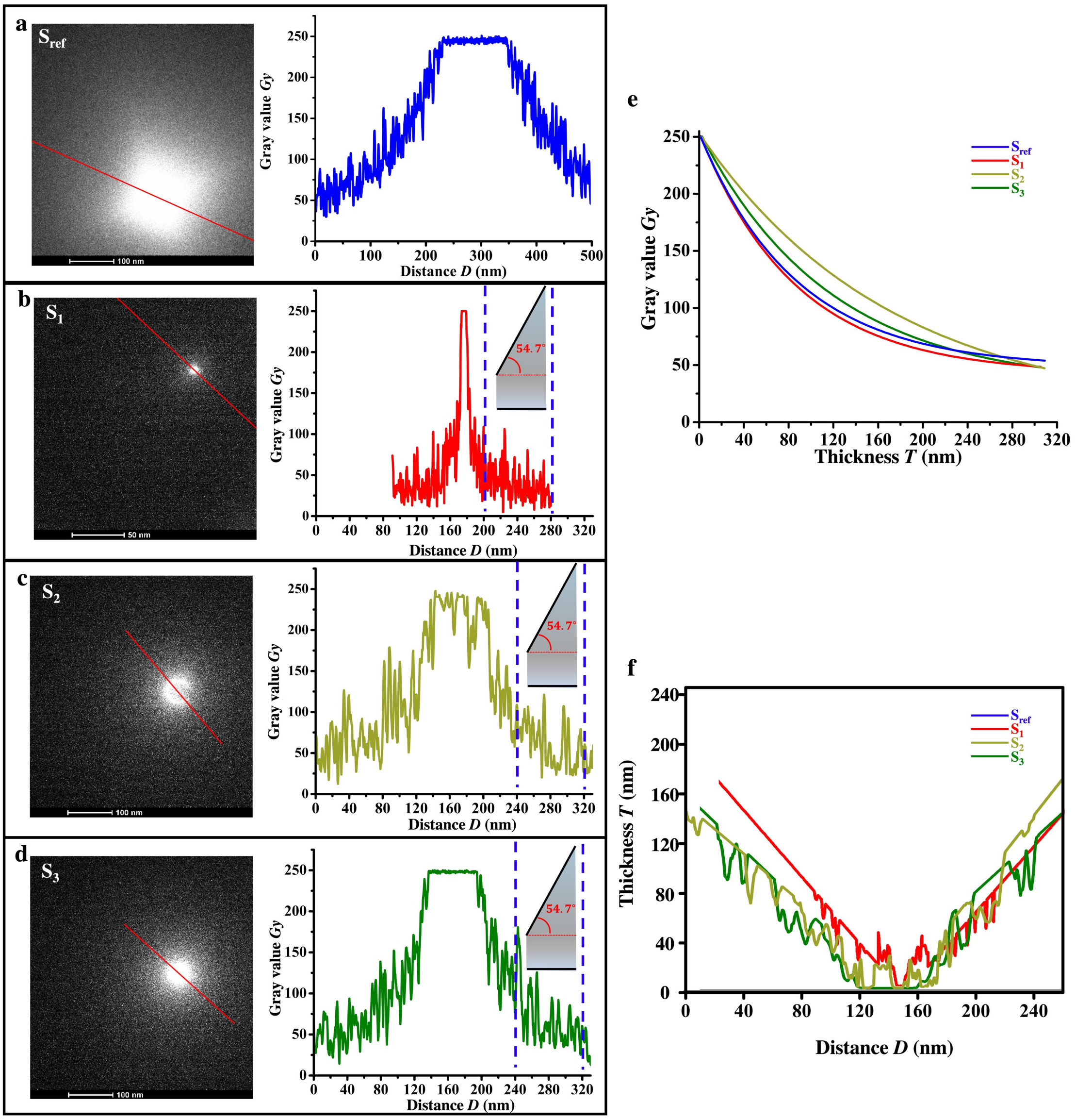

Figure S9. (a-d) Gray value (*Gy*) distributions along the red lines in the back-view TEM images of S_{ref}, S_1, S_2, and S_3. S_{ref} is a correction sample which has linearly etched sidewall in nanopore and known dependence of thickness (*T*) to distance (*D*), i.e., $\frac{\partial T}{\partial D} = tan54.7^\circ$. The curves in between two blue dashed lines in (b-d) correspond to the linearly etched sidewalls in MPSNs, demonstrating the same dependence of gray value (*Gy*) to distance (*D*) as that of S_{ref} and thus the same linear dependence of thickness (*T*) to distance (*D*). (e) The calibration curves, i.e., gray value (*Gy*) versus thickness (*T*), of S_{ref} (blue), S_1 (red), S_2 (gold), and S_3 (green). These calibration curves are derived by using the known dependences, i.e., $\frac{\partial T}{\partial D} = tan54.7^\circ$, $\frac{dGy}{dT} = -250ce^{-cT}$, and condition that *T* is 0 when *Gy* is 250. (f) Side-view nanopore profiles of S_1 (red), S_2 (gold), and S_3 (green), showing the curves of thickness (*T*) versus distance (*D*) derived using the calibration curves in (e).

The thickness and pore size of MPSN was determined using grayscale analysis. Figures S9a-d show the example gray value distributions along the red lines in the back-view TEM images of S_{ref}, S_1, S_2, and S_3. The direction of these red lines is parallel to <1,0,0> crystal orientation of the silicon samples. For crystal materials, the gray contrast of TEM image is sensitive to the material orientation[43], while the mass-thickness contrast keeps an approximate linearity based on Rutherford scattering theory under the condition of large collection semiangle of > 100 mrad[44]. This results in an exponential decay of the electron transmission intensity with thickness, i.e., $\frac{I_t}{I_i} = e^{-cT}$, where I_t and I_i are the transmission and incident intensity of electron

beam which are indicated by the gray value of TEM images, c is absorption coefficient determined by the atomic weight of sample and the cross section of elastic and inelastic scattering, T is the sample thickness.

For calculating the absorption coefficient c, a silicon nanopore sample S_{ref} of known etching thickness and linear structure feature was fabricated as correction sample. Here, the maximum Gray value of S_{ref} and nanopore sample is set to 250 (while the saturated level is 255) by adjusting the exposure time. Based on two known dependences, i.e., $\frac{\partial T}{\partial D} = tan54.7^\circ$, $\frac{dGy}{dT} = -250ce^{-cT}$, and condition that T is 0 when Gy is 250, the absorption coefficient c is calculated to be 0.028 ± 0.004 nm^{-1} according to the calibration curves of Gy verus T as shown in Fig. S9e. Then, the thickness T of samples can be described by measured Gy as:

$$T = -\frac{\ln Gy - 5.521}{0.028} \qquad \text{(S4)}$$

Hence, the thickness of nanopore samples can be accurately calibrated with the grayscale data distribution of S_{ref}, and the nanopore thickness profiles can be derived as shown in Fig. S9f. Further, the pore size ΔD_0 of samples can be described as:

$$\begin{cases} D = D_0, \text{while } Gy = 250 & \text{(S5)} \\ \Delta D_0 = \max(D_0) - \min(D_0) & \text{(S6)} \end{cases}$$

Supplementary Note 11. The repeatability study of the photovoltaic electrochemical etch-stop technique.

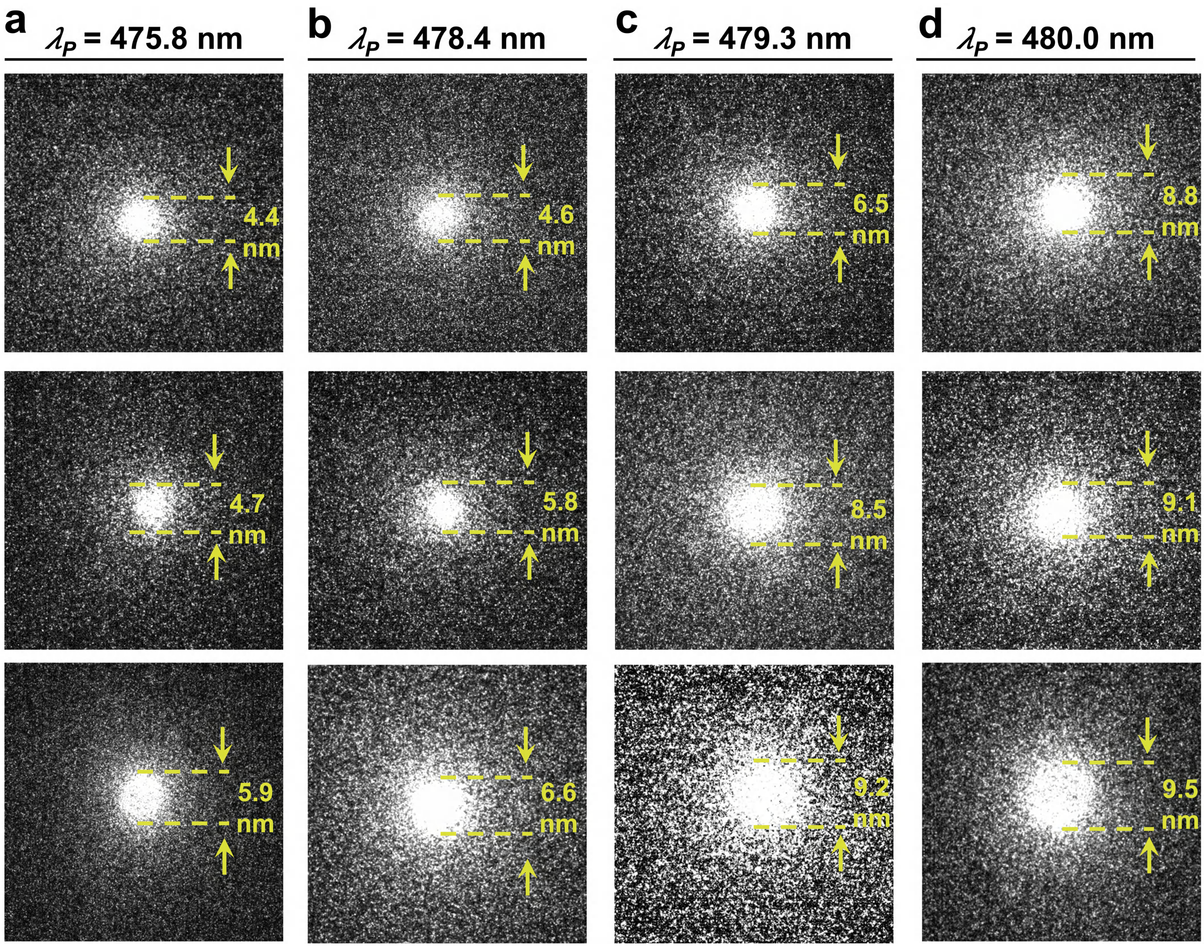

Figure S10. The TEM images of nanopores fabricated using over-etching time t_o of 0.6 seconds and the samples of variant remaining thicknesses (corresponding to different measured spectral peaks), i.e., H = 19±7 nm and λ_P = 475.8 nm (a), H = 76±8 nm and λ_P = 478.4nm (b), H = 95±8 nm and λ_P = 479.3 nm (c), H = 114±8 nm and λ_P = 480.0 nm (d).

Supplementary Note 12. Setting of the in-tube nanopore sensing device.

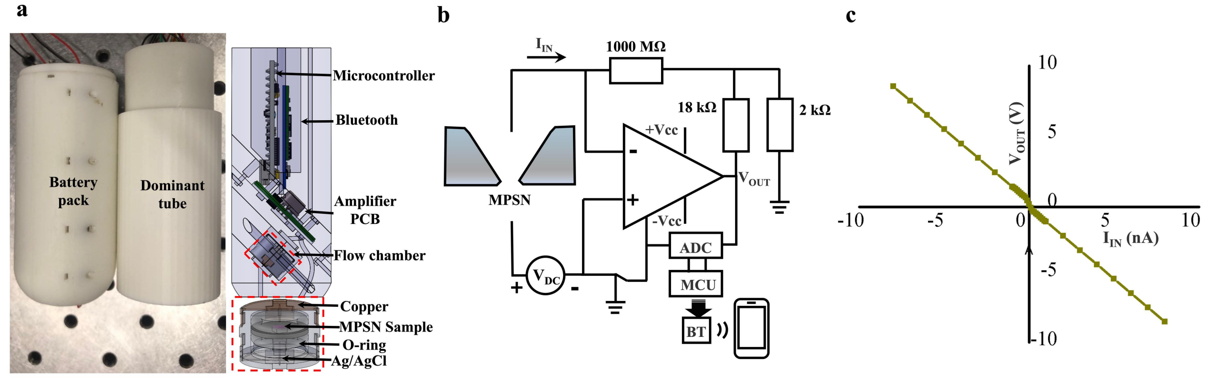

Figure S11. (a) Image (left) of in-tube nanopore sensing device, including a dominant tube and a battery pack. Schematic diagram (right) of internal detection modules in dominant tube. Inset within red dotted line shows the components of the flow chamber assembly, including Ag/AgCl electrodes, O-rings, coppers and the MPSN sample. (b) Simplified circuit diagram of the device. I_{IN} indicates the input current of the operational amplification circuit from current response of MPSN under applying potential V_{DC}. V_{OUT} is the output voltage of the operational amplification circuit. (c) The gain curve (V_{OUT}-I_{IN} curve) of the operational amplification circuit in (b).

Fig. S11c shows the gain curve between the output voltage V_{OUT} and the input current I_{IN} of the operational amplification circuit in Fig. S11b, which is used to amplify the blockade current signal. As the positive potential V_{DC} is connected to the negative input terminal of the operational transresistance amplifier, the gain characteristics can be described as:

$$V_{OUT} = -I_{IN}R_F \qquad (S7)$$

where R_F is the transresistance.

Supplementary Note 13. Molecular translocation under different pH values.

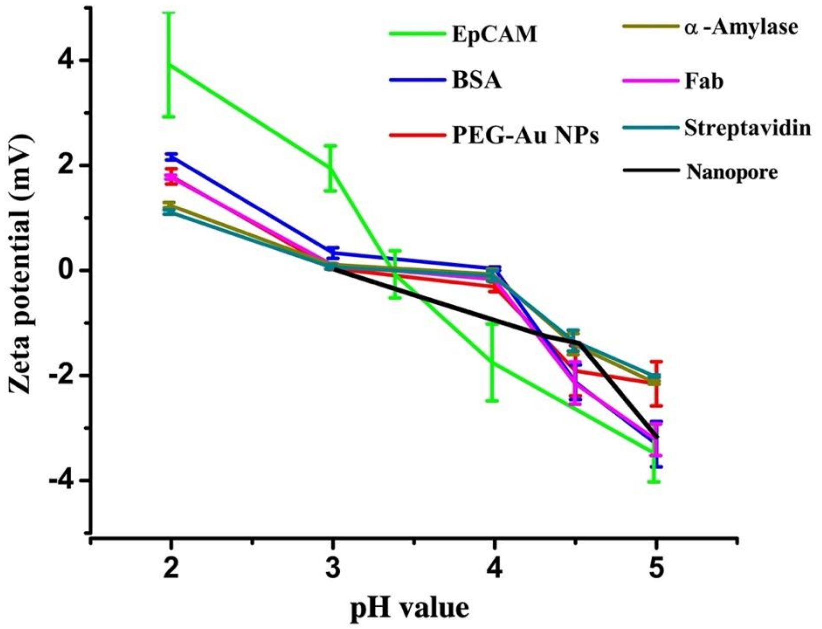

Figure S12. The function of zeta potential of different molecules and the MPSN to pH value. Error bars represent the measurement errors of the light scattering analyzer.

The dependence of Zeta potential on pH of the tested samples was tested with the DelsaMax PRO light scattering analyzer (Beckman Coulter), as shown in Fig. S12.

In general, the electrophoretic velocity v_{EP} of targets in the external electric field E is determined by ζ_{target} (i.e., the Zeta potential of target), while the electroosmotic flow velocity v_{EO} depends on $\zeta_{nanopore}$ (i.e., the Zeta potential of nanopore with charged sidewalls). Therefore, the effective velocity v_{tol} of the overall electric drive can be described as[45,46]:

$$v_{tol} = v_{EP} - v_{EO} = \frac{\varepsilon E}{\eta}(\zeta_{target} - \zeta_{nanopore}) \quad \text{(S8)}$$

where, ε is dielectric constant of solution, E is the intensity of electric field, and η is the solution viscosity.

Supplementary Note 14. Test the stability of MPSN.

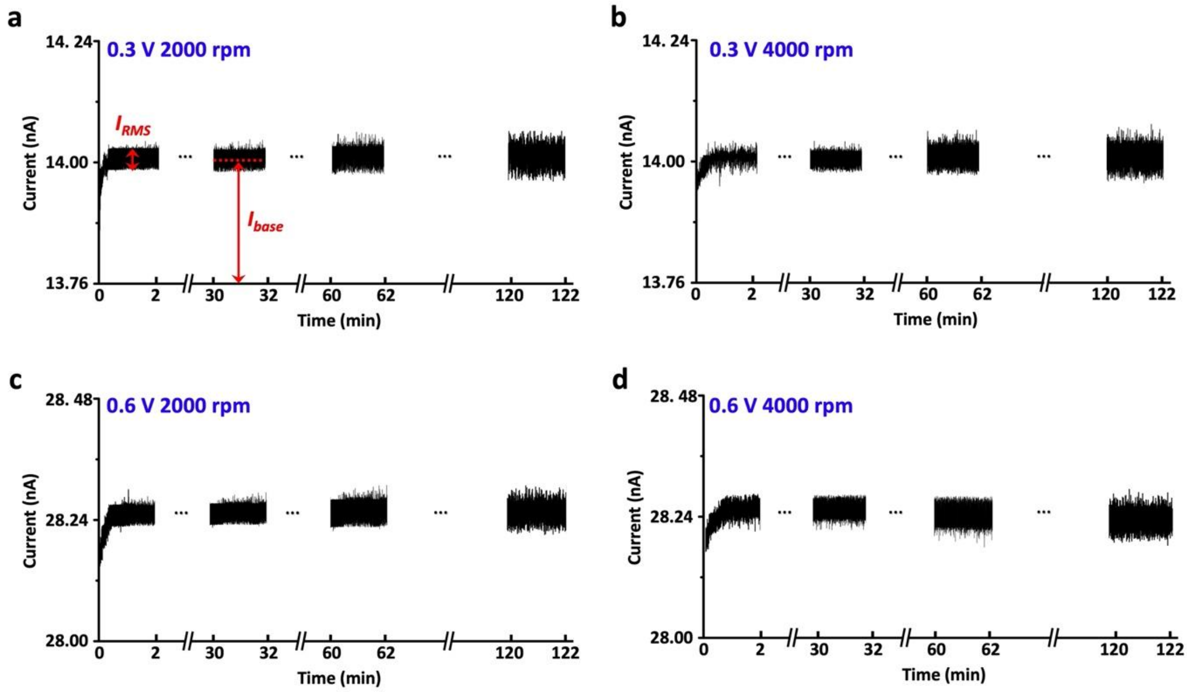

Figure S13. Current traces were obtained using 15-nm MPSN at different bias voltages and rotation speeds: 0.3 V and 2000 rpm (a), 0.3 V and 4000 rpm (b), 0.6 V and 2000 rpm (c), and 0.6 V and 4000 rpm (d), over a duration of more than 2 hours. Only selected signal episodes starting from 0, 30, 60, and 120 minutes are shown. I_{RMS} and I_{base} represent the root-mean-square current noise and current baseline, respectively. All measurements were conducted in a 1M KCl solution.

We conducted comprehensive tests on a 15-nm nanopore, varying the bias voltages and rotation speeds. Each test lasted for over 2 hours. Our results reveal that the applied voltages affect the current baseline (I_{base}), while the rotational speed has no impact. Furthermore, the root-mean-square current noise (I_{RMS}) remains independent of both bias voltage and rotation speed. Notably, when Ag/AgCl electrodes are immersed in the KCl solution, the current baseline (I_{base}) initially drifts due to transient polarization. However, it eventually stabilizes as a steady electrochemical interface forms between the electrode surface and the electrolyte[47]. Subsequently, the I_{RMS} of the current signal consistently increases from 45 to 95 pA over a 120-minute duration, under different test conditions. This systematic change in current noise is attributed to the amplified voltage noise of the operational amplifier[48].

Supplementary Note 15. Sensing area of MPSN.

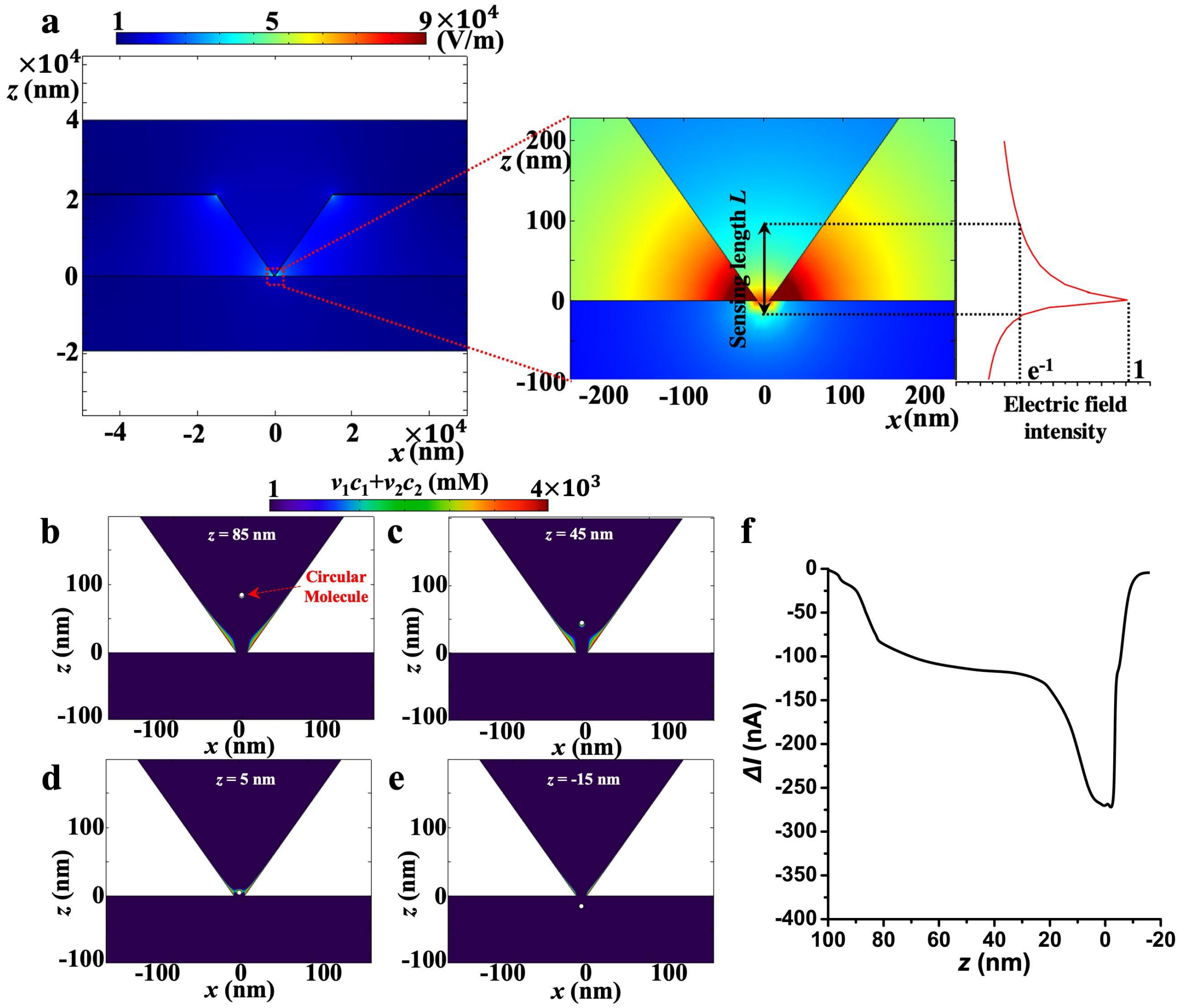

Figure S14. (a) The distribution of electric field in MPSN when the bias voltage is 0.3 V. The inset shows an enlarged view around the nanopore. The sensitive length L along the z direction is defined as the distance between the two points at which the electric field intensity is decayed to $e^{-1}$of the maximum intensity located in the nanopore centre. (b-e) Simulated translocation of a 7-nm circular molecule with surface charge of 17e through different locations at z = 85 (b), 45 (c), 5 (d), and -15 nm (e) by calculating spatial distribution of the net ionic concentration difference (v_1c_1+v_2c_2) in the MPSN within the area defined by x from -200 nm to 200 nm and z from -100 nm to 200 nm. Here, v_1 and v_2 represent the valences of potassium and chloride ions, respectively, while c_1 and c_2 represent the concentrations of potassium and chloride ions, respectively. (f) The corresponding current blockade change ΔI caused by the molecular translocation. The x- and z-direction are set to parallel to [0,1,0] and [1,0,0] crystal orientation of the silicon samples, respectively. The origin is positioned at the centre of the nanopore.

By implementing an ionic mass transport model in COMSOL, simulations were conducted to study the ion concentration change during the translocation of a circular molecule and therefore deduce the sensing area and length of MPSN (see Fig. S14a). The distribution of electrical field and the net ionic concentration were calculated using the Helmholtz Equation and Nernst-Planck-Poisson (NPP) equation, respectively. In the simulation, the electrolyte was 1M KCl, the nanopore material was single crystal silicon, and the pore size was set at 15 nm. The current signals were calculated using the conductivity equation of the electrolyte[49], which can be described as:

$$I = \int_S F(v_1\overrightarrow{N_1} + v_2\overrightarrow{N_2}) \cdot \vec{n} dS \qquad (S9)$$

where F is the Faraday constant; v_1 and v_2 represent the valences of potassium and chloride ions, respectively, while $\overrightarrow{N_1}$ and $\overrightarrow{N_2}$ represent the ionic flux of potassium and chloride ions, respectively; S is surface of the sensing area of the MPSN; $\vec{n}$ is outward unit normal vector.

By simulating the translocation of a 7-nm circular molecule with a surface charge of 17e through a nanopore, the study reveals that the current drop begins when the molecule's centre is positioned at z = 100 nm, and ends at -15 nm (see Fig. S14b-f).

Supplementary Note 16. Current traces of Au@PEG NPs under different pH values, rotation speeds, and applied voltages, and the dependence of capture rates on molecular concentration and rotation speed.

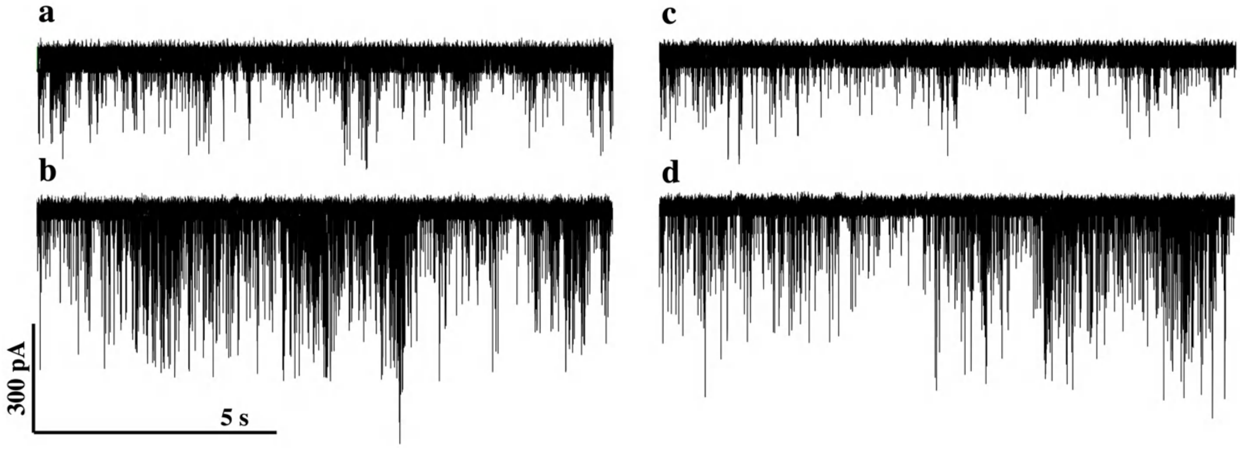

Figure S15. Electrokinetic current blockade traces of Au@PEG at voltages U of 0.3 V (a) and 0.6 V (b) and EpCAM at voltages of 0.3 V (c) and 0.6 V (d) at pH = 7.0 (nonequilibrium point).

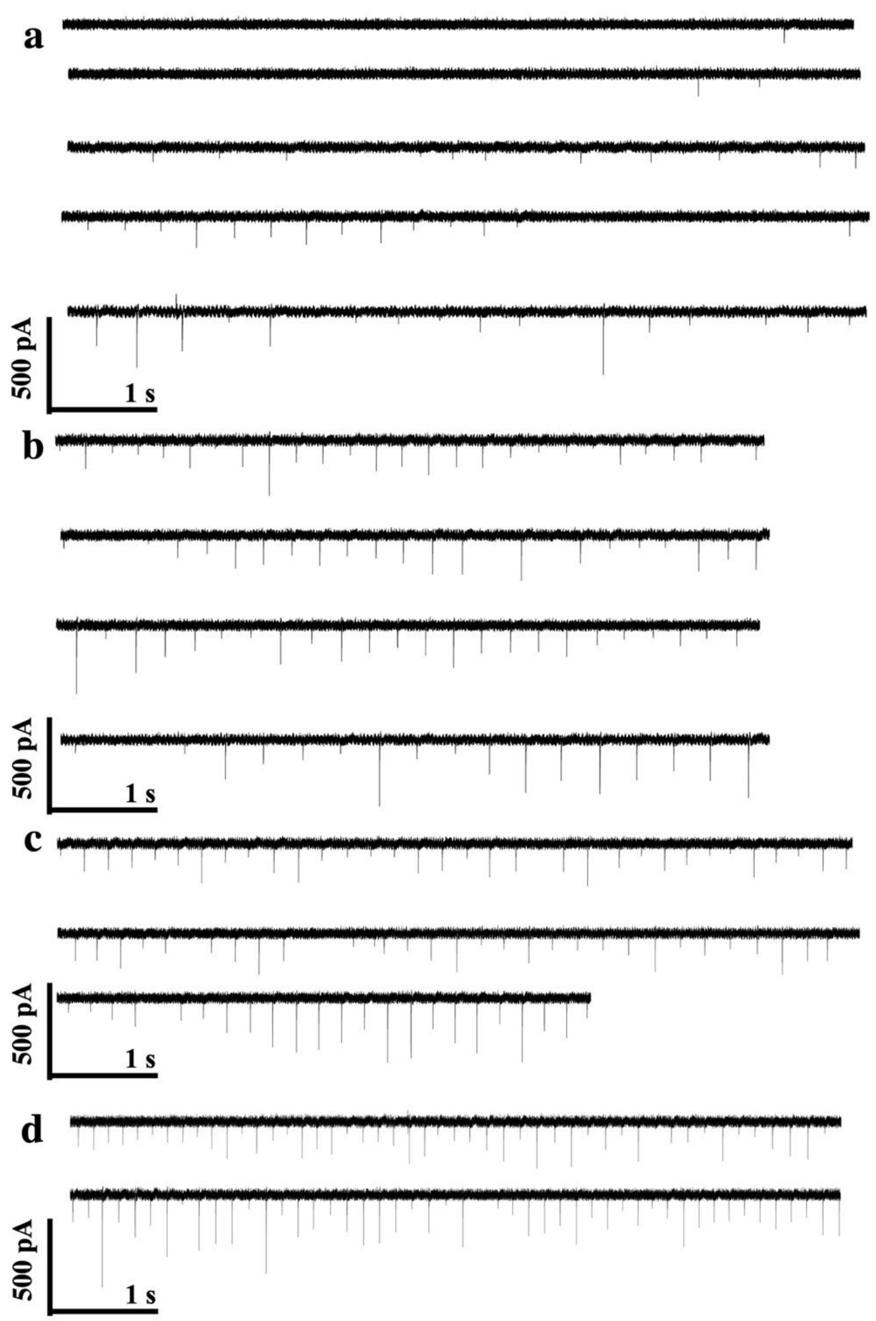

Figure S16. Inertial-kinetic current blockade traces of Au@PEG NPs at pH = 3.0 (equilibrium point) after 40 mins ultrasound measured at rotation speeds of 1000 (a), 2000 (b), 3000 (c), and 4000 rpm (d) at voltages of 0.3 V.

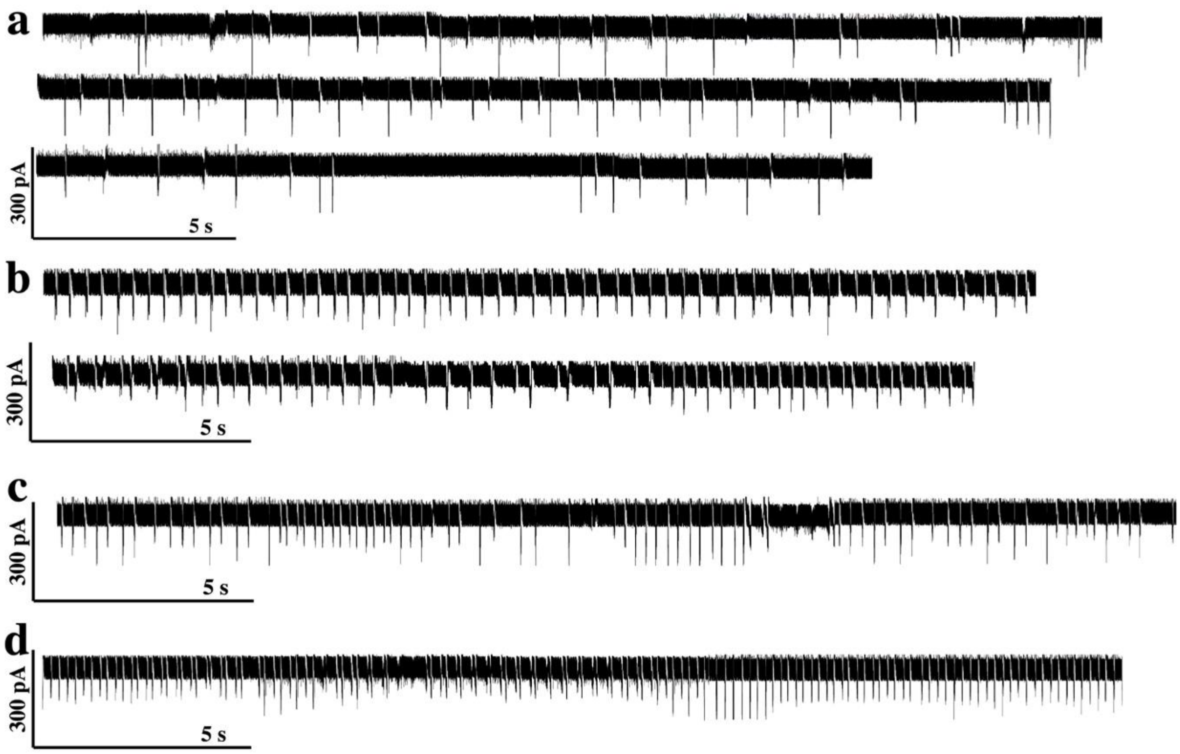

Figure S17. Inertial-kinetic current blockade traces of EpCAM after adjusting pH to 3.4 (equilibrium point) with rotation speed of 1000 (a), 2000 (b), 3000 (c), and 4000 rpm (d) at voltages of 0.3 V.

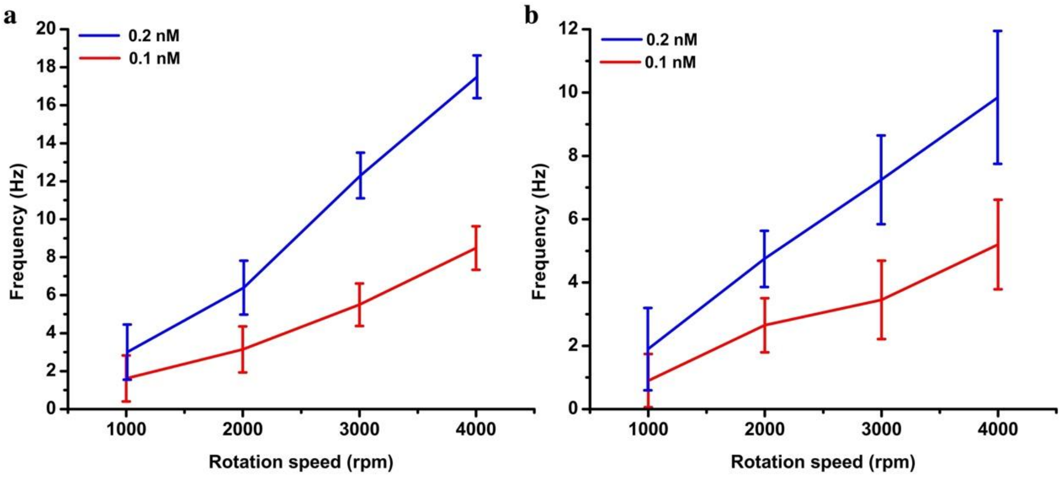

Figure S18. Capture rate of Au@PEG (a) and EpCAM (b) with different molecular concentration of 0.1 and 0.2 nM measured at different rotation speeds. Error bars represent the standard deviations of experimental data measured from the blockade signals.

The Figure S18 shows the increase of capture rate of Au@PEG and EpCAM with rotation speed. It is noted that both the capture rate at same rotation speed and its change of Au@PEG (m=500 kDa) is larger than these of EpCAM (m=37 kDa), although their molecular sizes are similar.

Supplementary Note 17. Compare the capture radius of the inertial-kinetic translocation with electrokinetic translocation in MPSN.

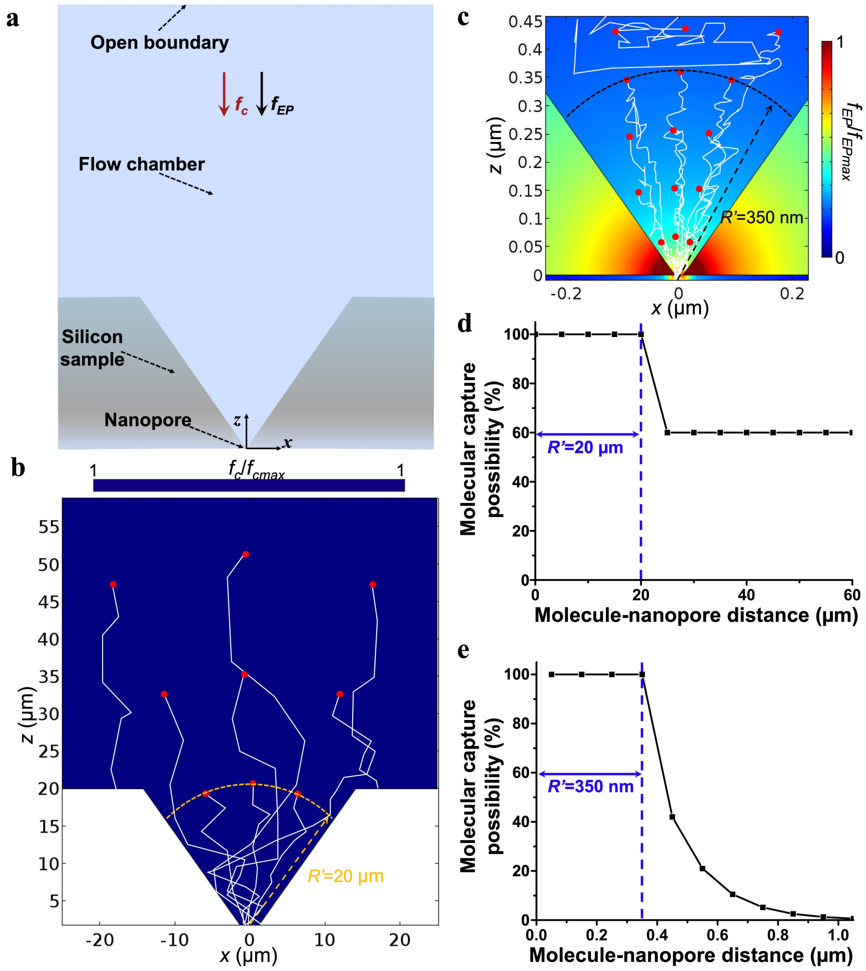

Figure S19. (a) Schematic of the model setup consisting of flow chamber, silicon sample, and nanopore. The upper boundary and the nanopore are set to open. The centrifugal force f_c and the electrophoretic force f_{EP} directed towards nanopores dominates the molecular behaviours in the inertial-kinetic translocation and the electrokinetic translocation in MPSN, respectively. The x- and z-direction are set to parallel to [0,1,0] and [1,0,0] crystal orientation of the silicon samples, respectively. The origin is positioned at the centre of the nanopore. Simulated translocation traces and capture radius of particle-like proteins in MPSN operated (b) under inertial force f_c and Brownian motion at a rotation speed of 4000 rpm and (c) under electrokinetic force f_{EP} and Brownian motion in 1M KCl solution. Red dots in (b) and (c) indicate the original locations of proteins with a molecular weight of 50 kDa, a diameter of 5 nm, and surface charge of 17e. f_{cmax} and f_{EPmax} denote the maximum centrifugal force and the maximum electrophoretic force, respectively. (d) and (e) show the dependence of molecular capture possibility on the molecule-nanopore distance under the inertial-kinetic translocation (d) and electrokinetic translocation (e). Here, the molecular capture possibility is defined as the ratio of molecular translocation events to all the molecular motion events, the molecule-nanopore distance is the length between the original location of the molecule and the nanopore centre, the capture radius is defined as the radial distance *R'* where the electrokinetic or the inertial-kinetic force starts to surpasses the diffusion-dominated dynamics[50]. In general, the molecular capture possibility is 100% while molecule-nanopore distance is within the capture radius *R'* of nanopore.

For electrokinetic translocation in a typical solid-state nanopore system, the molecular capture radius can be described as[50]:

$$R' = \frac{QU}{kT}\frac{D_p}{8+2\pi} \qquad \text{(S10)}$$

where Q is the surface charge of the trapped molecules (such as 17e for the BSA nominal charge at pH = 7)[51], U is the applied bias (i.e., 300 mV), *k* is the Boltzmann constant, T is the

environmental temperature, D_p is the pore size of the nanopore (i.e., 15 nm). Therefore, the capture radius of the nanopore to BSA is around 210 nm at pH = 7.

For the inertial-kinetic translocation in the MPSN, the molecular behaviour is influenced by the interplay between centrifugal force and Brownian diffusion. This competition determines the capture radius R' by comparing the relative magnitudes of molecular inertial potential energy and thermal energy. On the other hand, for the electrokinetic translocation in MPSN, the molecular capture radius R' can be determined by comparing the relative magnitudes of molecular electrophoretic force and local friction drag, and therefore the capture radius can be described as[50]:

$$R' = \frac{kT}{QE(R')} \qquad \text{(S11)}$$

where $E(R')$ is the electric field intensity at the capture radius R'.

To compare the capture radius (R') of two translocation methods in MPSN, a molecular motion model was developed using COMSOL Multiphysics. The model utilized the Langevin equation to study molecular traces. The simulation area ranged from x = -25 μm to 25 μm and z = 0 μm to 60 μm (see Fig S19a). A simulated protein with a molecular weight of 50 kDa, a diameter of 5 nm, and a surface charge of 17e was released within the simulated area (see Fig S19b and c). The simulation concluded when the protein exited the simulation area through translocation.

In the inertial-kinetic translocation simulation, the centrifugal force f_c is assumed to be constant throughout the chamber ($f_c/f_{cmax} = 1$) (see Fig S19b). The results demonstrate that the molecular translocation process is predominantly governed by inertial force, ensuring the proteins released within the pyramid structure with 20-μm thickness will be translocated through the nanopore. Thus, the capture radius R' of the inertial-kinetic translocation in the MPSN is around 20 μm[50].

As for the electrokinetic translocation, the electrophoretic force f_{EP} is calculated through simulating the distribution of electric field intensity E in MPSN (see Fig S19c). The results show that the molecular capture possibility is 100% when the distance between the starting location of molecule and the nanopore centre is within the capture radius, i.e., 350 nm, where the molecular behavior is dominated by the electrokinetic force. Compared with the electrokinetic translocation, the inertial-kinetic translocation provides a longer capture radius in MPSN (see Fig S19d and e).

Supplementary Note 18. The centrifugal force experienced by molecules at different rotational speeds in MPSN.

The centrifugal force f_c exerted on the molecules can be described as Equation S2. The centrifugal force experienced by representative molecules at different rotational speeds in MPSN is shown in Table S2. These calculated centrifugal forces are about 4-5 orders of magnitude larger than the collision forces between molecules under test and water molecules at room temperature (such as 10^{-12} to 10^{-10} pN for 50 kDa molecules).

Table S2. The centrifugal force f_c exerted on molecules of different molecular weight m at different rotational speeds ω.

Molecules	Amylase	BSA	Fab	Streptavidin	Au@PEG	EpCAM
Weight (kDa)	56	67.5	50	60	550	37
f_c at ω = 2000 rpm (pN)	1.0×10^{-6}	1.22×10^{-6}	9.02×10^{-7}	1.08×10^{-6}	9.93×10^{-6}	6.67×10^{-7}
f_c at ω = 4000 rpm (pN)	4.05×10^{-6}	4.87×10^{-6}	3.60×10^{-6}	4.34×10^{-6}	3.97×10^{-5}	2.67×10^{-6}

Supplementary Note 19. Mechanism of centrifugal-based molecular motions in MPSN

(a) Diffusion Coefficient $D(\beta)$.

Based on the Einstein-Smoluchowski Relation, the diffusion coefficient $D(\beta)$ of a molecule can be described as:

$$D(\beta) = \frac{kT}{f(\beta)} \qquad \text{(S12)}$$

where, k is the Boltzmann constant, T is the environmental temperature, and $f(\beta)$ is viscous drag coefficient, which is highly relative to the ratio of length to diameter β (Fig. S13b):

$$f(\beta) = 6\pi\mu RK' \qquad \text{(S13)}$$

where, R is the equatorial semi-axis of the molecules, and K′ is a shape factor. Notably, in the process of centrifugal drive, the molecules tend to turn to the direction of lower centrifugal potential energy. Thus, the K′ can be solely classified by β, as below[46]:

For a prolate ellipsoids,

$$K' = \frac{\frac{4}{3}(\beta^2 - 1)}{\frac{(2\beta^2 - 1)}{(\beta^2 - 1)^{\frac{1}{2}}}\ln\left[\beta + (\beta^2 - 1)^{\frac{1}{2}}\right] - \beta} \qquad \text{(S14)}$$

For an oblate ellipsoids,

$$K' = \frac{\frac{4}{3}((\beta^{-1})^2 - 1)}{\frac{(\beta^{-1})((\beta^{-1})^2 - 2)}{((\beta^{-1})^2 - 1)^{\frac{1}{2}}} arctan\left[((\beta^{-1})^2 - 1)^{\frac{1}{2}}\right] + (\beta^{-1})} \qquad \text{(S15)}$$

For a sphere,

$$K' = 1 \qquad \text{(S16)}$$

(b) Motion of molecules in capture process (ii)

In the process, the molecular behaviors are governed by competition between centrifugal force and Brownian diffusion. Thus, the molecular motion can be described as Langevin equation:

$$v(\beta) = \frac{1}{kT}D(\beta)f_e(\beta) + \sqrt{2D(\beta)}g(t) \qquad \text{(S17)}$$

where, $v_{(\beta)}$is velocity of the molecules, $f_e(\beta) = f_c(\beta)$, and $g(t)$ is the Gaussian noise term resulting from random collision forces. Substitute Equations S14-16 into the Langevin Equation S14, and the $v_{(\beta)}$ can be simplified as:

$$v(\beta) = \frac{f_c}{6\pi\mu RK'} \qquad \text{(S18)}$$

Thus, the duration t_1 of molecules in the process (time until) is

$$t_1 = \frac{6\pi\mu RK'L}{f_c} \qquad \text{(S19)}$$

(c) Motion of molecules in translocation-through-nanopore process (iii)

For each translocation event in the nanopore, the translocation time t_2 through nanopore (time until desorption) is a stochastic variable depending on the (bulk) dissociation rate k_d. Thus, we assumed an Eyring-like form[52]:

$$t_2 = t_0 e^{-h|fe|/kT} \qquad (S20)$$

where, the f_e on the molecule for its mass and shape appears in the exponent. The parameter h expresses the force-dependent factor, and it needs to be further modified by experimental data. t_0 is an exponential distribution with mean.

(d) Dependence of dwell time on molecular configuration

Then, the t_1, t_2 can be expressed as:

$$t_1 = \frac{6\pi\mu RK'L}{mr\omega^2} \qquad (S21)$$

where, ω is rotation speed (2000 and 4000 rpm) and ρ is the distance between the targets and the rotating shaft of the centrifuge (i.e. 25.6 cm for the system). β of majority of the tested molecules is between 0.5 and 2. Within this range, K' in Equation S21 can be simplified as:

$$K' = -1.378e^{-\frac{\beta}{0.762}} + 1.303 \qquad (S22)$$

Set shape-to-mass weighing factor (SMWF) $\xi = \frac{K'}{m}$, and then, substitute the SMWF ξ into Equations S21,

$$t_1 = \frac{6\pi\mu R\xi L}{\rho\omega^2} \qquad (S23)$$

The fitting results of Equations (2) and (3) lead to a sensing length of MPSN about 92 nm, which is close to the calculated value of 115 nm shown in Fig. S14.

Also, the translocation time t_2 can be expressed as:

$$t_2 = t_0 e^{-\frac{h(m\rho\omega^2)}{kT}} \qquad (S24)$$

The dependences shown in Equations (4) and (5) exhibit a force-dependent factor h is 8.45 µm.

Supplementary Note 20. Current traces for sensing the dissociation antibody-antigen complex and the aggregation of Au@PEG nanoparticles in 15-nm MPSN.

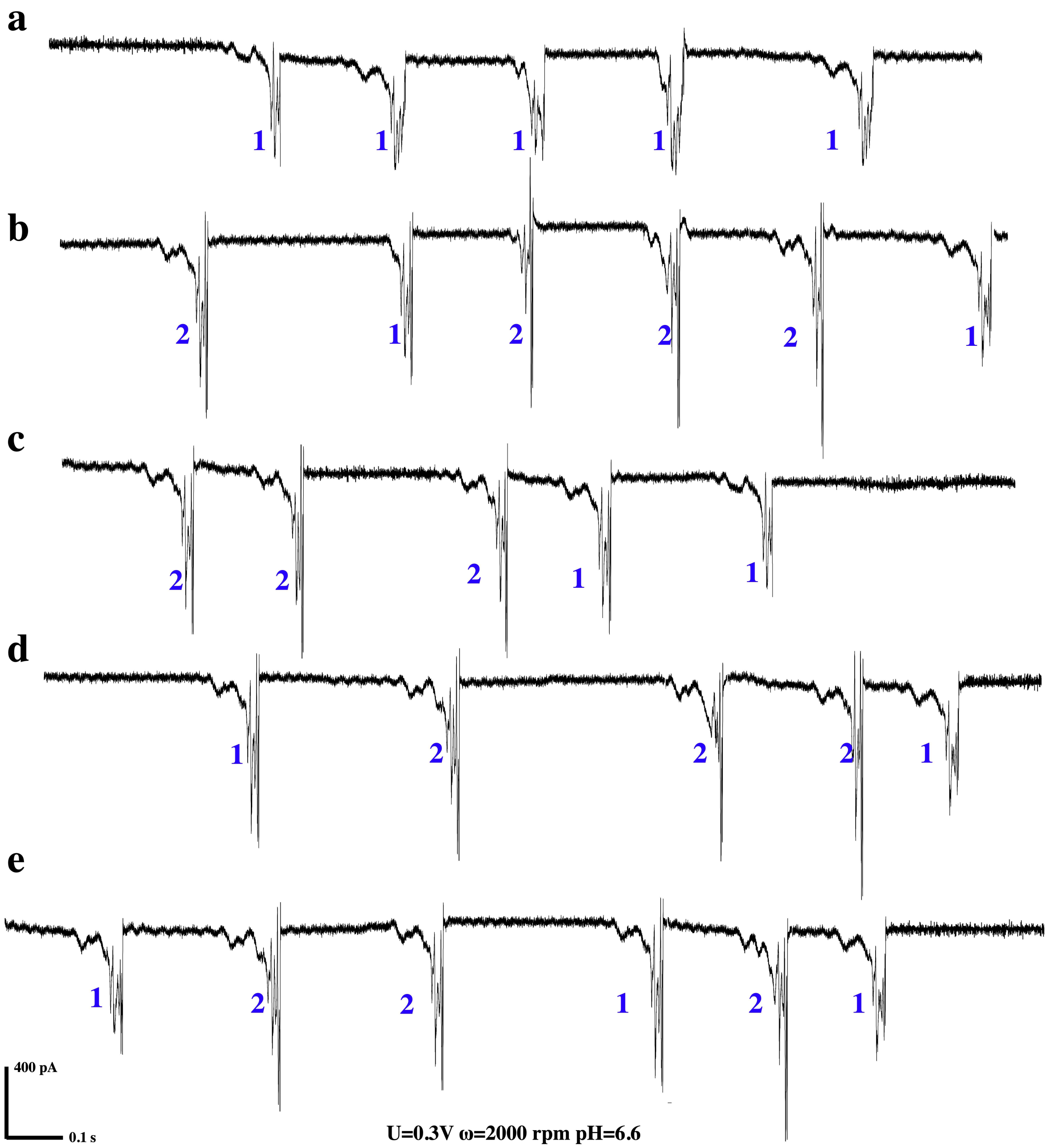

Figure S20. The current trajectory of EpCAM IgG not involved in the reaction (a) and mixture of EpCAM-antibody complex and IgG at 2 mins (b), 22 mins (c), 42 mins (d), and 62 mins (e) after adjusting pH to 6.6 (equilibrium point) with rotation speed of 2000 rpm at voltages of 0.3 V. The antibody and antibody-antigen complex signals in traces were labelled with 1 and 2, respectively.

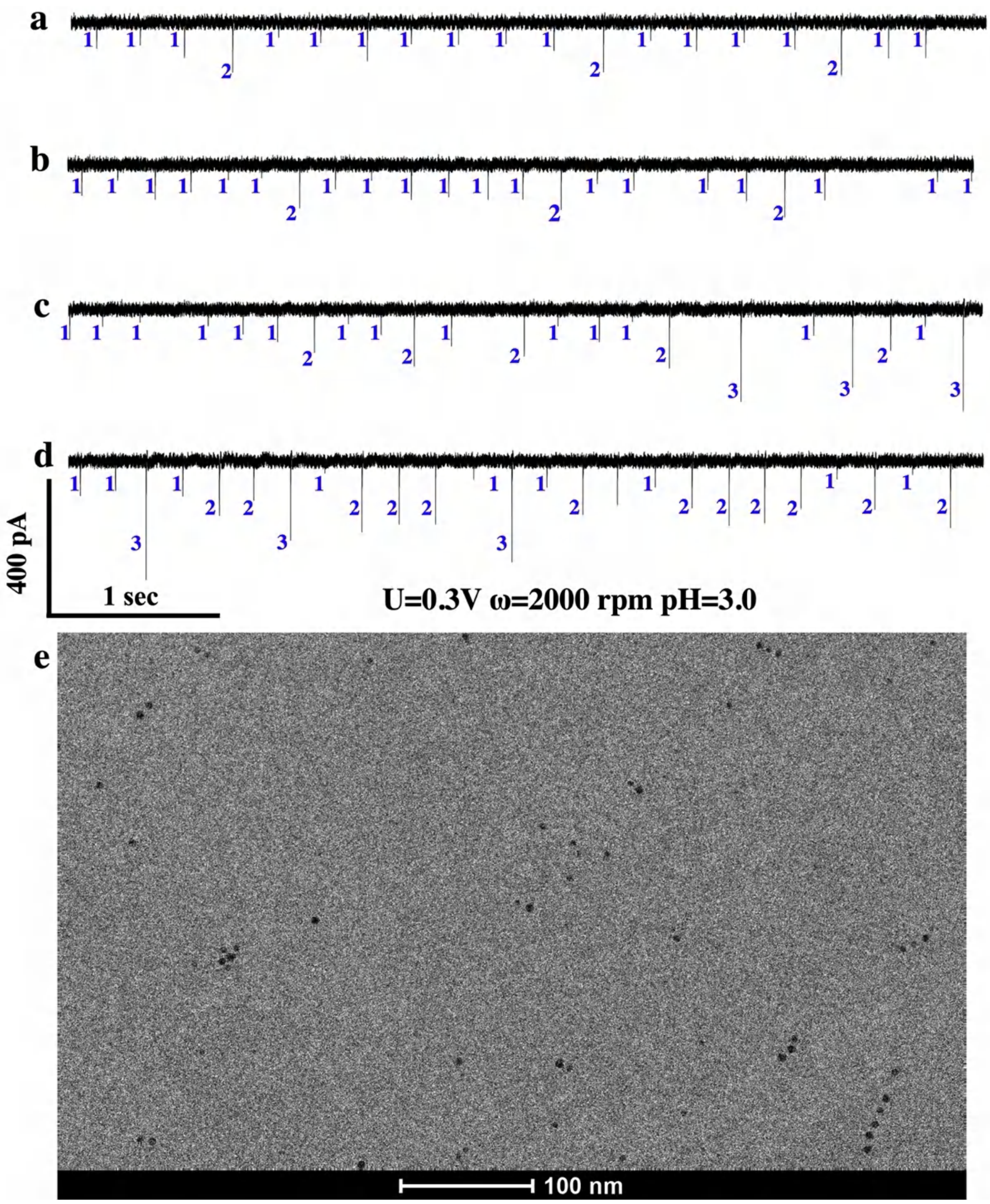

Figure S21. Current blockade traces of Au@PEG nanoparticel aggregations measured at 2 mins (a), 12 mins (b), 22 mins (c), and 32 mins (d) after the ultrasonic treatment. Current traces were measured at rotation speed of 2000 rpm and voltages bias of 0.3 V. The single molecule, bimolecules-aggregate, and trimolecules-aggregate signals in traces were labelled with 1, 2, and 3, respectively. (e) TEM image of Au@PEG nanoparticles. The sample is prepared at 32 mins after ultrasonic treatment.

Supplementary Note 21. Study the signal characteristics of EpCAM IgG and Au@PEG nanoparticles in trimolecular aggregate.

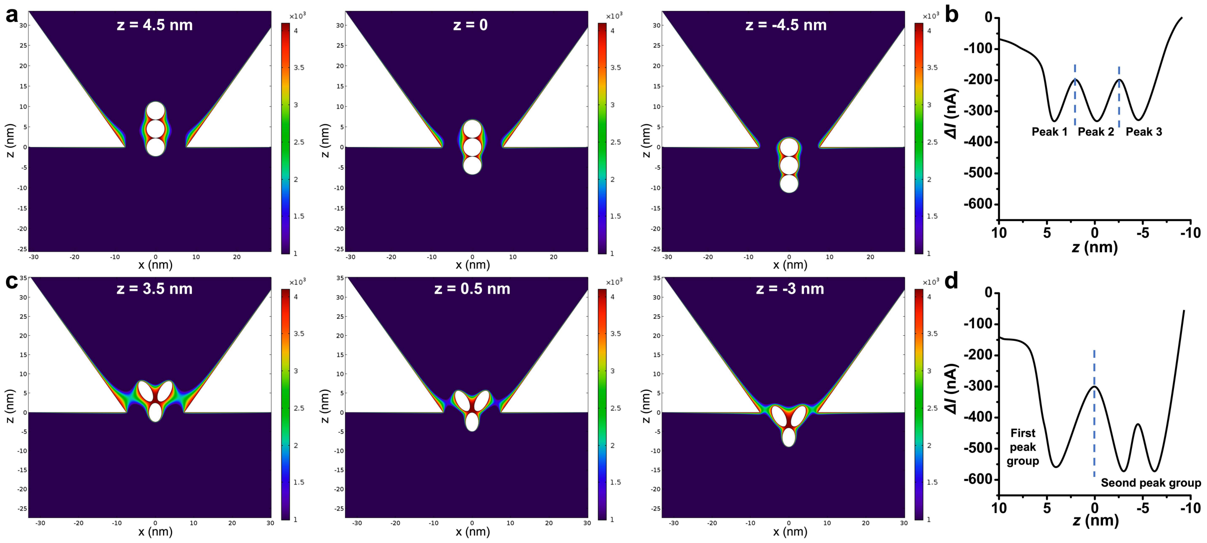

Figure S22. (a and c) Spatial distribution of the net ionic concentration difference ($v_1c_1+v_2c_2$) during the translocation of EpCAM IgG-like (a) and Au@PEG nanoparticles-like (c) trimolecular aggregates through a nanopore. Here, v_1 and v_2 represent the valences of potassium and chloride ions, respectively, while c_1 and c_2 represent the concentrations of potassium and chloride ions, respectively. (b and d) The corresponding current change ΔI associated with the EpCAM IgG-like (b) and Au@PEG nanoparticles-like (d) trimolecular aggregates.

To demonstrate the high conformational sensitivity of MPSN, we conducted simulations to observe the changes in ion concentration during the translocation of particle-like trimolecular aggregates through the sensing zone of MPSN. The simulation employed an ionic mass transport model implemented in COMSOL Multiphysics, where the mass transfer of ions and current density distribution were determined using the NPP equation[49]. The electrolyte used was 1M KCl, the nanopore sidewall was made of single crystal silicon, and the pore size was set at 15 nm. The simulated target molecules were EpCAM IgG-like trimolecular aggregates and Au@PEG nanoparticles-like trimolecular aggregates (see Fig. S22a and c). The corresponding feedback current signals ΔI were calculated using the conductivity equation of the electrolyte[49] (see Fig. S22b and d). The current signal of EpCAM IgG exhibited a two-peak-group characteristic, while the current signal of the Au@PEG trimolecular aggregate displayed a three-peak characteristic.

Supplementary Note 22. Current traces for sensing the dissociation of antibody-antigen complex in 20- and 23-nm MPSN.

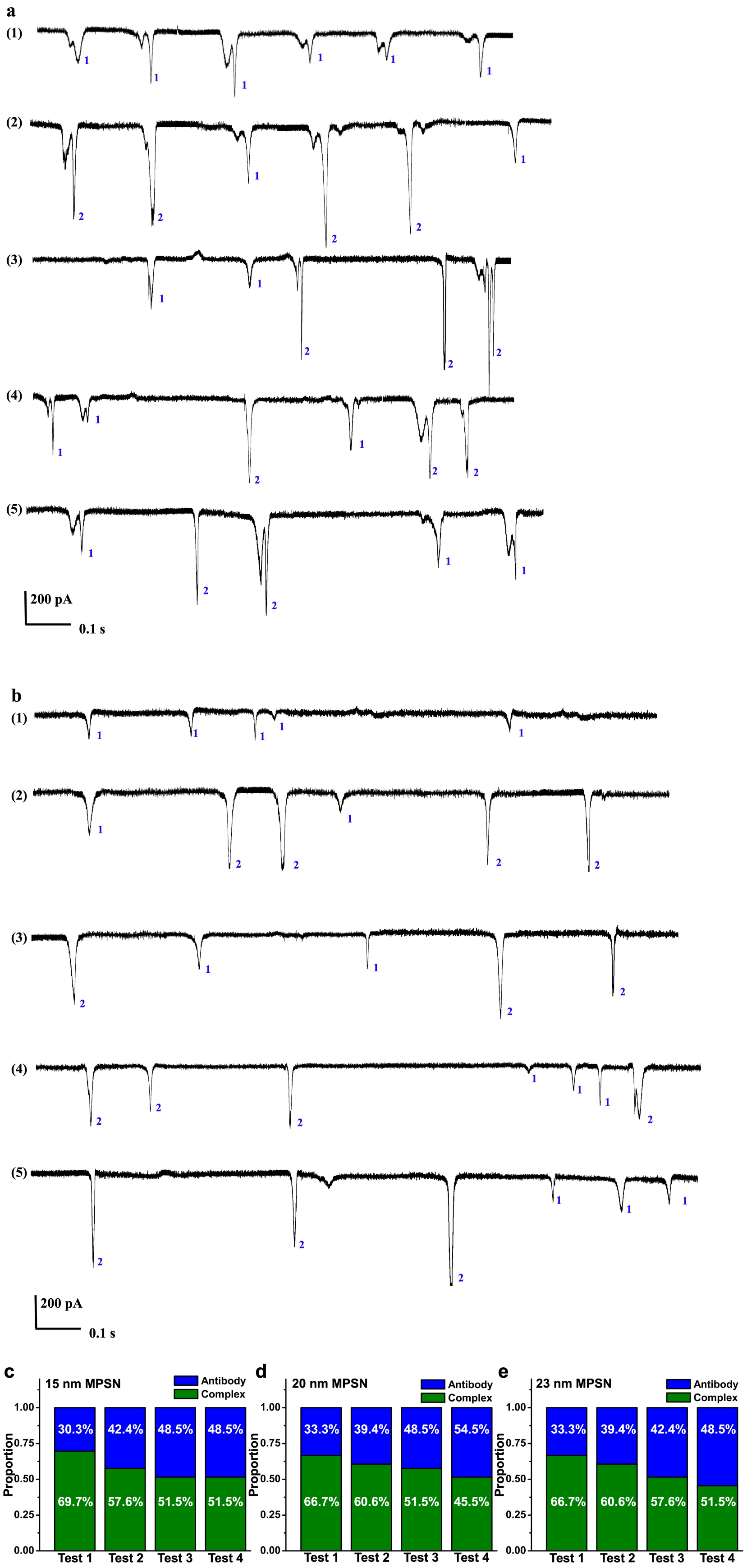

Figure S23. (a and b) Current traces obtained using 20-nm (a) and 23-nm (b) MPSNs. (1) represents the signal trace of EpCAM IgG alone, while (2-5) display the current traces of the EpCAM IgG-EpCAM antibody-antigen complex mixture measured at 2 mins, 22 mins, 42 mins, and 62 mins, respectively, after pH adjustment to 6.6 (the equilibrium point) at a rotation speed of 2000 rpm and bias voltages of 0.3 V. The antibody and antibody-antigen complex signals in the traces were labeled as "1" and "2," respectively. (c-e) Longitudinal monitoring of the dissociation dynamics of the antibody-antigen complex using 15-nm (c), 20-nm (d), and 23-nm (e) MPSNs, with four tests performed every 20 minutes.

To investigate the dissociation dynamics of the antibody-antigen complex, we conducted additional experiments using 20- and 23-nm MPSNs in addition to the 15-nm MPSN. The solution was found to have a complex ratio of 67.7%±1.8%, 59.6%±2.1%, 53.5%±3.5%, and 49.5%±4.3% measured at 2 mins, 22 mins, 42 mins, and 62 mins, respectively (see Fig. S23). Notably, the antibody translocation signal exhibited a single peak in both the 20- and 23-nm nanopores, while the characteristic multiple peak groups were observed only in the 15-nm nanopore.

Supplementary Note 23. Current traces of Au@PEGs aggregations purified using centrifugation-based separation protocol.

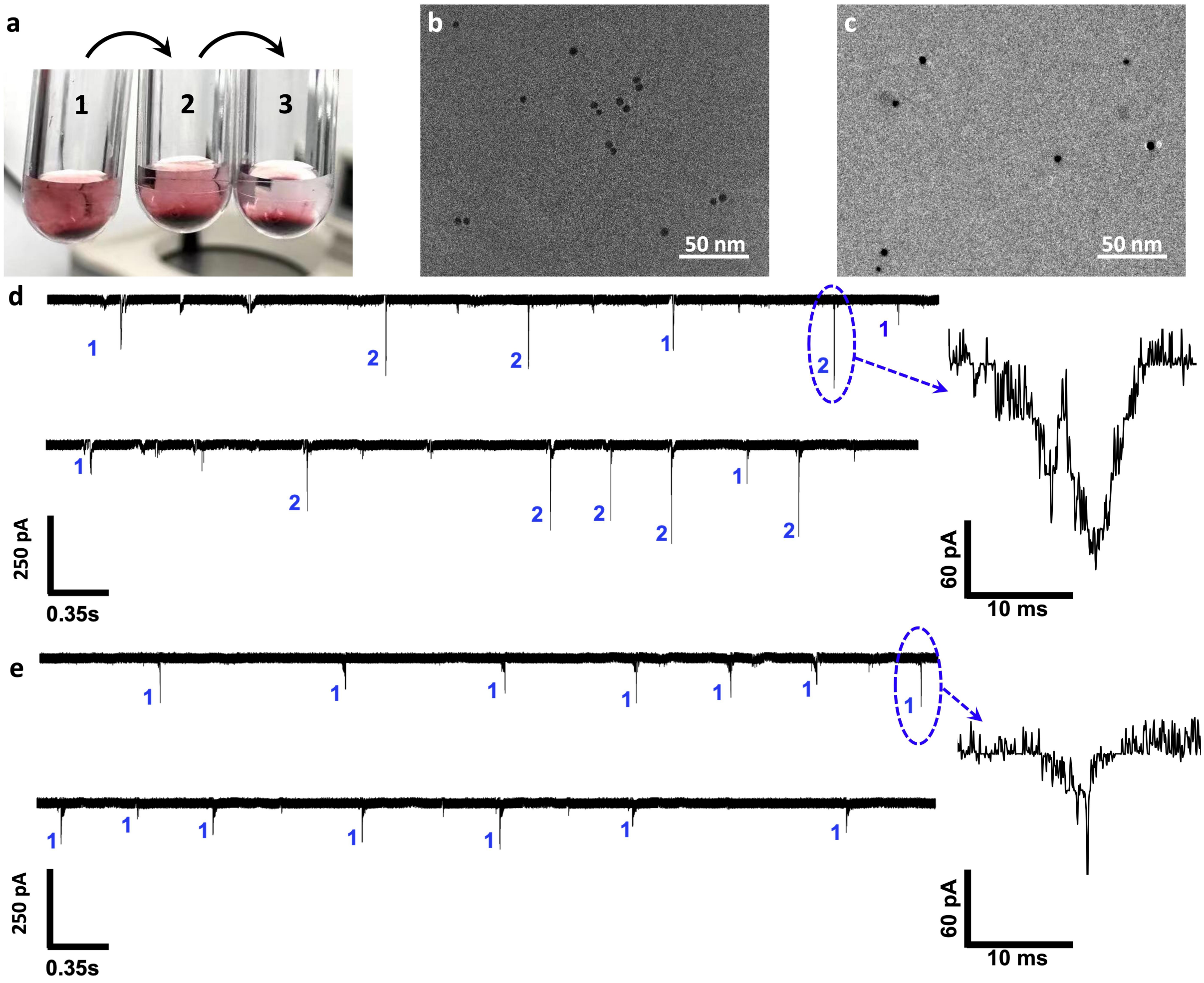

Figure S24. (a) 4.8-nm spherical Au@PEGs reagents after centrifugation at rotation speeds of 14000 (1), 16000 (2), and 18000 rpm (3) using the centrifugation-based separation protocol. (b) TEM image of a mixture containing 60% dimers and 40% monomers obtained from suspensions centrifugally purified at 16000 rpm. (c) TEM image of monomers obtained from suspensions centrifugally purified at 18000 rpm. (d and e) Current traces of suspensions purified at 16000 rpm (d) and 18000 rpm (e), measured at a rotation speed of 2000 rpm and a bias voltage of 0.3 V. Insets in (d) and (e) display representative two-peak and one-peak signals, respectively.

To confirm the correspondence between single-peak signals and single molecules, as well as double-peak signals and dimers, centrifugation-based separation protocol was employed to purify different aggregations[53]. The method involved systematically increasing the rotation speed to achieve stepwise g-force density centrifugation. After aggregating Au@PEGs at 92 °C, the samples were centrifuged at an initial speed of 10,000 rpm for 30 minutes. Subsequently, the rotation speed was increased by 2,000 rpm after each 30-minute centrifugation until reaching 20,000 rpm. The sediment was collected from the bottom of the centrifuge tube, and the supernatant was subjected to further centrifugation (see Fig. S24a).

At 16,000 rpm, a mixture comprising 60% dimers and 40% monomers was obtained (see Fig. S24b). Increasing the rotation speed to above 18,000 rpm resulted in almost 100% monomers (see Fig. S24c).

Furthermore, the suspensions purified at 16,000 rpm and 18,000 rpm were separately injected into the in-tube nanopore sensing system, enabling the acquisition of current blockade traces with inertial-kinetic translocation. The trace of the 16,000 rpm-purified suspensions exhibited

a ratio of two-peak singles at approximately 4:6 (see Fig. S24d), whereas all blockade signals of the 18,000 rpm-purified suspensions displayed the characteristic single peak (see Fig. S24e). This difference in ratio maps the single-peak signals to single molecules and the two-peak signals to biomolecule aggregates.

Supplementary References

1 Fürjes, P. Controlled focused ion beam milling of composite solid state nanopore arrays for molecule sensing. *Micromachines* **10**, 774 (2019).

2 Lo, C. J., Aref, T. & Bezryadin, A. Fabrication of symmetric sub-5 nm nanopores using focused ion and electron beams. *Nanotechnology* **17**, 3264 (2006).

3 Gadgil, V., Tong, H., Cesa, Y. & Bennink, M. L. Fabrication of nano structures in thin membranes with focused ion beam technology. *Surface and Coatings Technology* **203**, 2436-2441 (2009).

4 Sawafta, F., Carlsen, A. T. & Hall, A. R. Membrane thickness dependence of nanopore formation with a focused helium ion beam. *Sensors* **14**, 8150-8161 (2014).

5 Rudenko, M., Yin, D., Holmes, M., Hawkins, A. & Schmidt, H. in *Ultrasensitive and Single-Molecule Detection Technologies II.* 130-140 (SPIE).

6 Biance, A.-L. *et al.* Focused ion beam sculpted membranes for nanoscience tooling. *Microelectronic Engineering* **83**, 1474-1477 (2006).

7 Ibrahim, N. N. N. M. & Hashim, A. M. High sensitivity of deoxyribonucleic acid detection via graphene nanohole/silicon micro-nanopore structure fabricated by focused ion beam. *Materials Letters* **305**, 130740 (2021).

8 Komarov, F. *et al.* Ion-beam formation of nanopores and nanoclusters in SiO2. *Vacuum* **78**, 361-366 (2005).

9 Fu, Y., Bryan, N. K. A. & Fatt, L. T. Fabrication and characterization of nanopore array. *Journal of Nanoscience and Nanotechnology* **6**, 1954-1960 (2006).

10 Gierak, J. *et al.* Sub-5 nm FIB direct patterning of nanodevices. *Microelectronic Engineering* **84**, 779-783 (2007).

11 Morin, A. *et al.* FIB carving of nanopores into suspended graphene films. *Microelectronic Engineering* **97**, 311-316 (2012).

12 Md Ibrahim, N. N. N. & Hashim, A. M. Fabrication of Si Micropore and Graphene Nanohole Structures by Focused Ion Beam. *Sensors* **20**, 1572 (2020).

13 Waduge, P., Larkin, J., Upmanyu, M., Kar, S. & Wanunu, M. Programmed synthesis of freestanding graphene nanomembrane arrays. *Biophysical Journal* **108**, 330a (2015).

14 Wu, M.-Y., Krapf, D., Zandbergen, M., Zandbergen, H. & Batson, P. E. Formation of nanopores in a SiN/SiO2 membrane with an electron beam. *Applied Physics Letters* **87** (2005).

15 Kim, M. J., Wanunu, M., Bell, D. C. & Meller, A. Rapid fabrication of uniformly sized nanopores and nanopore arrays for parallel DNA analysis. *Advanced Materials* **18**, 3149-3153 (2006).

16 Van den Hout, M. *et al.* Controlling nanopore size, shape and stability. *Nanotechnology* **21**, 115304 (2010).

17 Graf, M. *et al.* Fabrication and practical applications of molybdenum disulfide nanopores. *Nature Protocols* **14**, 1130-1168 (2019).

18 Feng, J. *et al.* Single-layer MoS2 nanopores as nanopower generators. *Nature* **536**, 197-200 (2016).

19 Rigo, E. *et al.* Measurements of the size and correlations between ions using an electrolytic point contact. *Nature Communications* **10**, 2382 (2019).

20 Storm, A., Chen, J., Ling, X., Zandbergen, H. & Dekker, C. Fabrication of solid-state nanopores with single-nanometre precision. *Nature Materials* **2**, 537-540 (2003).

21 Zeng, S., Wen, C., Solomon, P., Zhang, S.-L. & Zhang, Z. Rectification of protein translocation in truncated pyramidal nanopores. *Nature Nanotechnology* **14**, 1056-1062 (2019).

22 Kwok, H., Briggs, K. & Tabard-Cossa, V. Nanopore fabrication by controlled dielectric breakdown. *PLOS One* **9**, e92880 (2014).

23 Wang, Y., Chen, Q., Deng, T. & Liu, Z. Self-aligned nanopore formed on a SiO2 pyramidal membrane by a multipulse dielectric breakdown method. *The Journal of Physical Chemistry C* **122**, 11516-11523 (2018).
24 Wang, Y., Chen, Q., Deng, T. & Liu, Z. Nanopore fabricated in pyramidal HfO2 film by dielectric breakdown method. *Applied Physics Letters* **111** (2017).
25 Arcadia, C. E., Reyes, C. C. & Rosenstein, J. K. In situ nanopore fabrication and single-molecule sensing with microscale liquid contacts. *ACS Nano* **11**, 4907-4915 (2017).
26 Bandara, Y. N. D., Karawdeniya, B. I. & Dwyer, J. R. Push-button method to create nanopores using a tesla-coil lighter. *ACS omega* **4**, 226-230 (2019).
27 Lan, W.-J., Holden, D. A., Zhang, B. & White, H. S. Nanoparticle transport in conical-shaped nanopores. *Analytical chemistry* **83**, 3840-3847 (2011).
28 Steinbock, L. J., Bulushev, R. D., Krishnan, S., Raillon, C. & Radenovic, A. DNA translocation through low-noise glass nanopores. *ACS Nano* **7**, 11255-11262 (2013).
29 Chen, K. *et al.* Super-Resolution Detection of DNA Nanostructures Using a Nanopore. *Advanced Materials* **35**, 2207434 (2023).
30 Holden, D. A., Hendrickson, G., Lyon, L. A. & White, H. S. Resistive pulse analysis of microgel deformation during nanopore translocation. *The Journal of Physical Chemistry C* **115**, 2999-3004 (2011).
31 Alawami, M. F. *et al.* Lifetime of glass nanopores in a PDMS chip for single-molecule sensing. *Iscience* **25** (2022).
32 Deng, T., Chen, J., Wu, C. & Liu, Z. Fabrication of inverted-pyramid silicon nanopore arrays with three-step wet etching. *ECS Journal of Solid State Science and Technology* **2**, P419 (2013).
33 Park, S. R., Peng, H. & Ling, X. S. Fabrication of nanopores in silicon chips using feedback chemical etching. *Small* **3**, 116-119 (2007).
34 Chen, Q., Wang, Y., Deng, T. & Liu, Z. Fabrication of nanopores and nanoslits with feature sizes down to 5 nm by wet etching method. *Nanotechnology* **29**, 085301 (2018).
35 Strandman, C. & Backlund, Y. Bulk silicon holding structures for mounting of optical fibers in V-grooves. *Journal of Microelectromechanical Systems* **6**, 35-40 (1997).
36 Voss, R., Siedel, H. & Baumgartel, H. in *TRANSDUCERS'91: 1991 International Conference on Solid-State Sensors and Actuators. Digest of Technical Papers.* 140-143 (IEEE).
37 Vaz, M. A. *et al.* Experimental and numerical analyses of the ultimate compressive strength of perforated offshore tubular members. *Marine Structures* **58**, 1-17 (2018).
38 Chong, D. Y., Lee, W., Lim, B., Pang, J. H. & Low, T. in *The Ninth Intersociety Conference on Thermal and Thermomechanical Phenomena In Electronic Systems (IEEE Cat. No. 04CH37543).* 203-210 (IEEE).
39 Gaspar, J., Paul, O., Chu, V. & Conde, J. Mechanical properties of thin silicon films deposited at low temperatures by PECVD. *Journal of Micromechanics and Microengineering* **20**, 035022 (2010).
40 Park, S., Lim, J., Pak, Y. E., Moon, S. & Song, Y.-K. A solid state nanopore device for investigating the magnetic properties of magnetic nanoparticles. *Sensors* **13**, 6900-6909 (2013).
41 Wanunu, M. *et al.* Rapid electronic detection of probe-specific microRNAs using thin nanopore sensors. *Nature Nanotechnology* **5**, 807-814 (2010).
42 Wen, C., Zhang, Z. & Zhang, S.-L. Physical model for rapid and accurate determination of nanopore size via conductance measurement. *ACS Sensors* **2**, 1523-1530 (2017).
43 Wei, R., Pedone, D., Zürner, A., Döblinger, M. & Rant, U. Fabrication of metallized nanopores in silicon nitride membranes for single-molecule sensing. *Small* **6**, 1406-1414 (2010).

44 Zhang, H.-R., Egerton, R. F. & Malac, M. Local thickness measurement through scattering contrast and electron energy-loss spectroscopy. *Micron* **43**, 8-15 (2012).
45 Firnkes, M., Pedone, D., Knezevic, J., Doblinger, M. & Rant, U. Electrically facilitated translocations of proteins through silicon nitride nanopores: conjoint and competitive action of diffusion, electrophoresis, and electroosmosis. *Nano Letters* **10**, 2162-2167 (2010).
46 Matijevic, E. *Medical Applications of Colloids*. (Springer, 2008).
47 Cranny, A. & Atkinson, J. K. Thick film silver-silver chloride reference electrodes. *Measurement Science and Technology* **9**, 1557 (1998).
48 Rosenstein, J. K., Wanunu, M., Merchant, C. A., Drndic, M. & Shepard, K. L. Integrated nanopore sensing platform with sub-microsecond temporal resolution. *Nature Methods* **9**, 487-492 (2012).
49 Yeh, L.-H., Zhang, M., Qian, S., Hsu, J.-P. & Tseng, S. Ion concentration polarization in polyelectrolyte-modified nanopores. *The Journal of Physical Chemistry C* **116**, 8672-8677 (2012).
50 Qiao, L., Ignacio, M. & Slater, G. W. Voltage-driven translocation: Defining a capture radius. *The Journal of Chemical Physics* **151** (2019).
51 Kubiak-Ossowska, K., Jachimska, B., Al Qaraghuli, M. & Mulheran, P. A. Protein interactions with negatively charged inorganic surfaces. *Current Opinion in Colloid & Interface Science* **41**, 104-117 (2019).
52 Chinappi, M., Yamaji, M., Kawano, R. & Cecconi, F. Analytical model for particle capture in nanopores elucidates competition among electrophoresis, electroosmosis, and dielectrophoresis. *ACS Nano* **14**, 15816-15828 (2020).
53 Novak, J. P., Nickerson, C., Franzen, S. & Feldheim, D. L. Purification of molecularly bridged metal nanoparticle arrays by centrifugation and size exclusion chromatography. *Analytical Chemistry* **73**, 5758-5761 (2001).
